# Advancing preclinical research with reconstructed in vitro skin models mimicking non-healing wounds

**DOI:** 10.1016/j.ijpx.2026.100518

**Published:** 2026-03-17

**Authors:** Regina Gomes Daré, Luciana B. Lopes, Alke Petri-Fink, Barbara Rothen-Rutishauser

**Affiliations:** a Institute of Biomedical Sciences, University of São Paulo, 1524 Professor Lineu Prestes Avenue, 05508-000 São Paulo, Brazil; b Adolphe Merkle Institute, University of Fribourg, Chem. des Verdiers 4, 1700 Fribourg, Switzerland; c Department of Chemistry, University of Fribourg, Chemin du Musée 9, 1700 Fribourg, Switzerland

**Keywords:** Chronic wounds, Pharmacological targets, Human skin equivalents, Reconstructed human epidermis, Tissue engineering

## Abstract

Chronic skin wounds remain a significant therapeutic challenge worldwide, primarily due to persistent inflammation, impaired function of fibroblasts and keratinocytes, defective angiogenesis, and the presence of complex polymicrobial biofilms. Conventional animal models only partially capture these human-specific pathophysiological mechanisms, limiting their predictive value for pharmacological development. Recent advances in human 3D in vitro skin models, including reconstructed human epidermis, full-thickness skin equivalents, vascularized and innervated constructs, and chronic wound–derived cell systems, provide opportunities to evaluate therapeutic strategies under controlled, human-relevant conditions. Here, we critically synthesize how engineered skin platforms recreate key pathological hallmarks of non-healing wounds, including IL-1/TNF-α–driven inflammation, RAGE–NOX4-mediated oxidative stress, MMP/TIMP imbalance, fibroblast and keratinocyte senescence, impaired HIF-1α/VEGF-dependent angiogenesis, immune polarization defects, and biofilm-associated antimicrobial tolerance. We examine scaffold-based, decellularized, and bioprinted approaches that enable the incorporation of adipocytes, endothelial cells, sensory neurons, and immune compartments, enhancing the mechanistic resolution with which chronic wound biology can be interrogated. By integrating cellular, biochemical, immune, vascular, and microbial components, next-generation models allow pharmacological interrogation of targets such as IL-1/IL-1R, IL-6/STAT3, TNF-α/TNFR, RAGE–NOX4, Nrf2/KEAP1, ERK/AKT, Ang/Tie2, ferroptosis regulators, senescence pathways, and neuroimmune modulators. Collectively, these platforms bridge the gap between reductionist assays and clinical complexity, offering a rational framework for mechanism-based drug discovery and preclinical screening. This review provides guidelines for selecting and designing advanced human skin models to accelerate the development of effective therapeutics for chronic non-healing wounds.

## Highlights

• Human 3D skin models reproduce the chronic wound microenvironment beyond acute injury.
• Advanced skin equivalents enable mechanism-based screening of wound therapeutics.
• Human-relevant models improve preclinical screening beyond conventional animal assays.
• Model complexity can be tailored to specific drug development questions.
• Ideal multicellular platforms integrate immune, vascular, neural and microbial components.

## Graphical abstract

**Figure:**
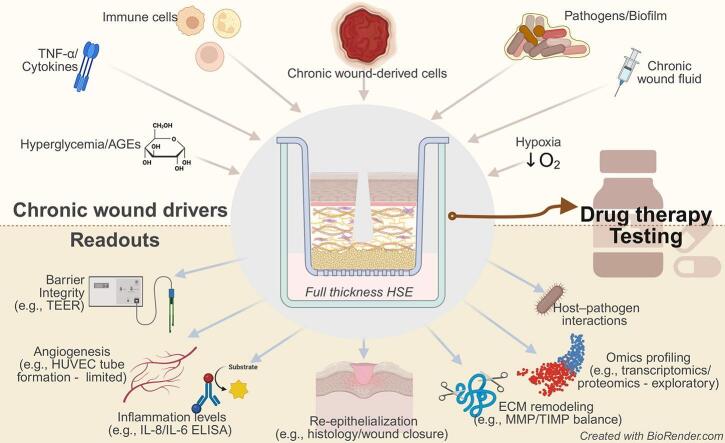

## 1 Introduction

Chronic wounds, also known as non-healing wounds, remain a major clinical challenge, affecting millions of patients worldwide and imposing substantial socioeconomic burdens (GBD, 2021). Their pathophysiology is multifactorial, involving persistent inflammation, impaired angiogenesis, cellular senescence, protease-driven matrix degradation, and microbial biofilms (Hofmann et al., 2023a), which together limit the efficacy of conventional therapies. Although animal models have supported therapeutic development (Avci et al., 2013), differences in skin architecture and repair mechanisms, such as wound closure by contraction in rodents versus re-epithelialization in humans (Avci et al., 2013; Bédard et al., 2020; Hofmann et al., 2023b), alongside ethical and regulatory pressures, constrain their predictive value (Bédard et al., 2020).

Human-based in vitro skin models offer a compelling alternative. Advances in scaffold engineering, bioprinting, and stem cell technologies have enabled the reconstruction of increasingly complex three-dimensional (3D) human skin equivalents that incorporate epithelial stratification, dermal–epidermal crosstalk, and extracellular matrix (ECM) organization (Catarino et al., 2018; Hofmann et al., 2023b; Pedrosa et al., 2017). Beyond structural fidelity, these models can be modularly adapted to integrate pathological drivers such as hyperglycemia, immune dysfunction, and polymicrobial colonization (Hofmann et al., 2023a; Quílez et al., 2024), thereby improving pathophysiological relevance.

Despite progress, there is currently no comprehensive pharmacological framework that integrates the mechanistic dysregulation of chronic wounds with the capabilities of human 3D skin models. Existing reviews focus primarily on bioengineering innovations or clinical aspects, but do not critically examine how human-relevant models reveal targetable pathways, including interleukin-1 and its receptor (IL-1/IL-1R), tumor necrosis factor alpha and its receptor (TNF-α/TNFR), interleukin-6 and signal transducer and activator of transcription 3 signaling (IL-6/STAT3), receptor for advanced glucation end products and NADPH oxidase 4 (RAGE–NOX4), imbalance between matrix metalloproteinases and their tissue inhibitors (MMP/TIMP), hypoxia-inducible factor-1-alpha (HIF1α)-driven vascular dysfunction, ferroptosis, senescence, and immune polarization defects (Bédard et al., 2020; Hofmann et al., 2023a; Cioce et al., 2024; Fernández-Guarino et al., 2023).

Here, we provide a mechanism-focused review that synthesizes how human 3D skin models, including full-thickness constructs, decellularized dermis, bioprinted tissues, vascularized systems, and chronic-wound–derived cell models, enable the evaluation of therapeutic candidates with improved translational fidelity. By integrating pathophysiology, model engineering, and therapeutic testing, this review aims to guide the rational selection, design, and pharmacological application of next-generation skin models for the development of chronic wound drugs.

## 2 From 2D monolayers to 3D architectures

Over the past few decades, it has become evident that simple 2D culture models of the dominant cell types in the skin (i.e., keratinocytes or fibroblasts) fail to replicate physiological conditions due to the lack of cell-cell and cell-matrix interactions (Sun et al., 2006). Keratinocyte monolayers, for instance, fail to develop hemidesmosomes, keratohyalin granules, or gap junctions, and typically exhibit deficient expression of high-molecular-weight keratins (Pruniéras et al., 1983). They also differ from their in vivo counterparts in cellular morphology, gene expression patterns, proliferation rates, migration behavior, and responses to external stimuli (Antoni et al., 2015).

**Fig. 1:**
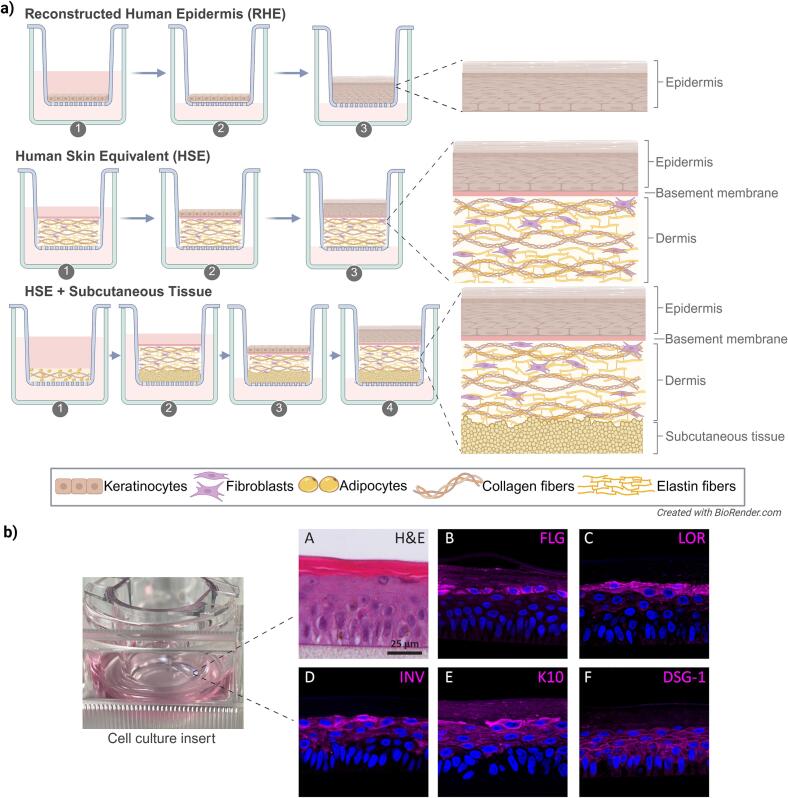
Generation of 3D reconstructed human skin models and representative characterization of Reconstructed Human Epidermis (RHE). (a) Three main processes for generating 3D in vitro reconstructed human skin models. RHE: Step 1. Keratinocyte seeding: Primary keratinocytes are seeded onto porous inserts submerged in culture medium. Step 2. Proliferation and confluence (2-3 days). Cells form a confluent monolayer under submerged conditions. Step 3. Air-liquid Interface (ALI). Inserts are raised to the ALI to induce keratinocyte stratification and differentiation. Maturation. A multi-layered epidermis with a well-defined stratum corneum forms over 10–14 days. Human Skin Equivalent (HSE): Step 1. Dermal preparation: Fibroblasts are embedded in a dense hydrogel matrix (e.g., collagen type I and elastin fibers) and polymerized in inserts (2–3 days). Step 2. Keratinocyte seeding (24-72 h post-polymerization): Keratinocytes are added atop the dermal matrix. Step 3. ALI: Constructs are raised to ALI to promote epidermal differentiation. Maturation: Around 10–14 days. HSE with subcutaneous tissue: Step 1. Subcutaneous layer preparation (adipogenesis; 1-2 weeks): Pre-differentiated adipocytes or adipose-derived stromal cells are embedded in a soft hydrogel matrix (e.g., collagen type I) for lipid accumulation. Step 2. Dermal preparation: Fibroblasts in a dense hydrogel matrix are layered atop the subcutaneous compartment (2–3 days). Step 3. Keratinocyte seeding (24-72 h): Keratinocytes are seeded on the dermal surface. Step 4. ALI: The construct is raised to ALI to promote epidermal differentiation. Maturation: Around 10–14 days. Created in https://BioRender.com. (b) Representative images of RHE. Left: macroscopic view of a cell culture insert containing RHE. (A) Histological analysis (H&E staining) showing a multi-layered stratified epidermis. (B—F) Confocal immunofluorescence images demonstrating expression of epidermal differentiation and adhesion markers: (B) filaggrin (FLG), (C) loricrin (LOR), (D) involucrin (INV), (E) keratin 10 (K10), and (F) desmoglein 1 (DSG-1) (magenta). Nuclei are counterstained with DAPI (blue). Scale bar = 25 μm. Panel (A–F) adapted from Dijkhoff et al. (2021) with permission under the Creative Commons Attribution, NonCommercial, NoDerivs 3.0 Unported License. (For interpretation of the references to colour in this figure legend, the reader is referred to the web version of this article.)

To overcome these limitations, 3D in vitro skin models have been developed as physiologically relevant platforms for studying skin biology, disease pathophysiology, and therapeutic response. These models span increasing levels of complexity: (i) reconstructed human epidermis (RHE), composed solely of stratified keratinocytes; (ii) full-thickness human skin equivalents (HSE), which integrate keratinocytes and fibroblasts to simulate both epidermal and dermal layers; and (iii) tri-layered constructs incorporating a subcutaneous adipose layer (Fig. 1).

An advancement in epidermal modeling came with the development of the air-liquid interface (ALI) culture system, originally established in the early 1980s by Pruniéras et al. (1983), which enabled keratinocytes to stratify when cultured on permeable supports. Commercial insert systems (e.g., Transwell®, Millicell®) facilitate basolateral nutrient supply while exposing the apical surface to air, thereby promoting barrier formation and differentiation (Handler et al., 1989; Poumay and Coquette, 2007). A basement-membrane substitute, typically composed of type I or IV collagen, further supports adhesion and maturation (Pruniéras et al., 1983). Although ALI culture enhances lipid barrier formation, additional biochemical cues are required for terminal differentiation. Supplementation with calcium (∼1.2 mM) induces keratin K1/K10, involucrin, filaggrin, and loricrin expression (Bikle et al., 2012; Hennings et al., 1980), whereas epidermal growth factor (EGF), keratinocyte growth factor (KGF), and ascorbic acid further enhance stratification and lipid barrier formation (Andreadis et al., 2001; Hashimoto, 2000; Ponec et al., 1997). Such refinements underpin regulatory-validated RHE models (e.g., EpiSkin®, SkinEthic® RHE, EpiDerm®), which are used for irritation and corrosion assays in compliance with OECD guidelines (OECD, 2021, OECD, 2019). A more comprehensive overview of the available models is given by Wever et al. (2013).

The development of HSE models represents a significant advancement. These systems incorporate a dermal compartment of fibroblasts embedded in a matrix, typically collagen-based, overlaid with keratinocytes cultured at ALI (Bell et al., 1991). Crosstalk between fibroblasts and keratinocytes, mediated by paracrine factors such as KGF, granulocyte-macrophage colony-stimulating factor (GM-CSF), and IL-6, drives basement membrane deposition, epidermal stratification, and terminal differentiation beyond that achievable in keratinocyte-only systems (Smola et al., 1993). Furthermore, fibroblasts actively remodel the ECM, increasing matrix density and mechanical strength, which promotes more physiologically relevant keratinocyte differentiation (Bell et al., 1991). Validated commercial HSEs, such as EpiDermFT™ and the Phenion® Skin Models, are increasingly applied in regulatory testing.

Recent innovations aim to extend physiological fidelity by adding a subcutaneous adipose layer. These tri-layered constructs begin to capture the structural and biochemical influence of adipocytes on skin homeostasis. Yet, the in vitro maintenance of mature adipocytes remains challenging: they are fragile, buoyant, and prone to dedifferentiation. Scaffold-based strategies can prolong adipocyte survival, but sustaining a functional phenotype over extended culture remains an ongoing challenge (Dufau et al., 2021).

Importantly, the transition from 2D to 3D systems has profound pharmacological implications. Keratinocytes and fibroblasts in 2D monolayers fail to recapitulate signaling pathways relevant to chronic wound drug responses, including calcium-dependent terminal differentiation (Hennings et al., 1980; Bikle et al., 2012), epidermal–mesenchymal crosstalk mediated by KGF, GM-CSF, and IL-6 (Smola et al., 1993), and structural cues regulating migration and proliferation (Antoni et al., 2015). As a result, therapeutic responses observed in 2D often overestimate efficacy or mask key mechanisms. In contrast, 3D HSEs reproduce barrier maturation, lipid organization, cytokine-driven inflammatory activation, and ECM remodeling, processes required for evaluating anti-inflammatory drugs, MMP inhibitors, pro-migratory agents, and immunomodulators under relevant conditions (Poumay and Coquette, 2007; Bell et al., 1991; Ponec et al., 1997). These advantages position 3D models as superior platforms for predicting therapeutic performance more reliably than 2D cultures.

## 3 Skin tissue engineering approaches

To approximate the structural and functional complexity of native human skin, researchers have employed diverse engineering strategies in developing reconstructed 3D skin models. These approaches differ primarily in their use of materials and techniques to support cell growth, tissue organization, and functional maturation. The following section will provide an overview of the technologies currently employed in skin model development.

### 3.1 Keratinocyte and fibroblast sources

The biological relevance of engineered skin models is linked to the choice of cell sources. Primary human keratinocytes and fibroblasts, isolated from surgical discards, biopsies, or commercial biobanks, remain the benchmark for their physiological fidelity in recapitulating native tissue architecture and function in vitro (Quílez et al., 2024). Donor age is a key determinant: neonatal keratinocytes exhibit robust proliferation and minimal donor-to-donor variability, enabling reproducible generation of stratified epidermal equivalents, whereas adult keratinocytes capture age-associated phenotypes such as reduced proliferative capacity and enhanced inflammatory signaling (Chin et al., 2023). A similar trend is observed for fibroblasts, with neonatal cells supporting matrix deposition and epithelial–mesenchymal crosstalk, while adult fibroblasts display senescence-associated features that alter tissue stiffness and organization (Fullard et al., 2024; Nan et al., 2025).

**Fig. 2:**
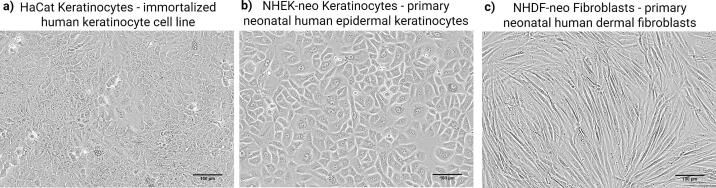
Phase contrast light microscopy images of immortalized and primary human skin cell sources that can be employed in engineered skin models. a) Immortalized human keratinocyte cell line (HaCaT), used for its scalability and interlaboratory reproducibility, despite presenting phenotypic drift over time. b) Primary neonatal human epidermal keratinocytes (NHEK-neo), characterized by high proliferative potential, low donor-to-donor variability, and capacity to generate fully stratified epidermal equivalents. c) Primary neonatal human dermal fibroblasts (NHDF-neo), exhibiting robust proliferative activity. Scale bar = 100 μm.

While the use of primary cells can be constrained by limited tissue availability and replicative senescence, particularly for in-house isolations, these challenges are ameliorated by sourcing cryopreserved, quality-controlled cells from biobanks, which enhances experimental standardization and reproducibility (Fig. 2). As an alternative, immortalized cell lines (e.g., HaCaT keratinocytes) offer advantages in scalability and interlaboratory comparability. However, their utility is often limited by genetic and phenotypic drift, which can compromise the accuracy of physiological and pathological responses (Drasler et al., 2017).

Recent immortalization strategies have yielded next-generation keratinocyte models with improved differentiation capacity. The N/TERT1 and N/TERT2G lines, immortalized via human telomerase reverse transcriptase (hTERT) expression, demonstrate ability to form stratified epithelia that closely mimic primary cell-derived models in terms of morphology, barrier integrity, and differentiation marker expression (Smits et al., 2017). Similarly, the NHK-E6/E7 line, utilizing HPV oncogenes, has been shown to generate well-differentiated epidermal equivalents under ALI culture (Jahn et al., 2024). Parallel progress has been achieved with dermal fibroblasts, where hTERT-immortalized lines such as BJ-5ta (CRL-4001) (Szymański et al., 2020) have been integrated into collagen scaffolds to generate human skin equivalents. In this setting, BJ-5ta populated the dermal matrix, expressed vimentin, and contributed to cytokine secretion, supporting the establishment of a functional full-thickness model.

### 3.2 Scaffold-based strategies for dermal reconstruction

Chronic wound pathogenesis, driven by persistent inflammation, impaired vascularization, and microbial colonization (see section 4 for details), demands scaffold strategies that surpass structural support to actively modulate these pathological cues. Scaffolds serve as a structural foundation for 3D skin models, providing both physical and biochemical cues for cell attachment, proliferation, and tissue maturation. Translating scaffold innovations into clinically relevant wound models requires careful consideration of material properties (e.g., biodegradability, stiffness) and microarchitecture (e.g., porosity, fiber alignment). These requirements guide the design of three main scaffold types: (i) matrix-based scaffolds (e.g., soft hydrogels), which prioritize bioactivity; (ii) structured scaffolds (e.g., pre-formed porous materials), which enhance mechanical stability; (iii) decellularized dermal scaffolds, which preserve native ECM complexity.

#### 3.2.1 Matrix-based scaffolds

Hydrogel-based scaffolds dominate current skin modeling because they closely mimic the native ECM, supporting cell-matrix interactions, nutrient diffusion, and tissue remodeling. Natural polymers (e.g., collagen and fibrin) excel in biocompatibility but suffer from batch variability and weak mechanics, whereas synthetic alternatives (e.g., poly(ethylene gly*co*l) (PEG), poly(ε-caprolactone) (PCL), and polylactic-co-glycolic acid (PLGA)) provide tunability but lack innate bioactivity (Elizabeth E Antoine et al., 2014; Khan et al., 2024).

Collagen type I remains the gold standard for dermal reconstruction, accounting for approximately 90% of the connective tissue proteins in the skin. Derived primarily from rat tail tendon or bovine skin, collagen hydrogels offer excellent biocompatibility and low immunogenicity (Chevallay and Herbage, 2000). However, its performance is highly concentration-dependent: concentrations below 4 mg/mL lack mechanical stability, while those exceeding 20 mg/mL create overly dense networks that impede cell migration. Gelation kinetics and matrix architecture are further influenced by temperature, pH, and ionic strength during polymerization (Elizabeth E. Antoine et al., 2014).

#### 3.2.2 Structured scaffolds

To address the mechanical limitations of soft hydrogels, structured scaffolds employ pre-formed porous materials (synthetic or natural) to provide stability, resistance to contraction, and long-term culture. Natural options, such as hyaluronic acid fiber networks, can support sustained ECM deposition and epidermal stratification for weeks (Stark et al., 2006), whereas synthetic polymers, including PCL, PLGA, and PEG derivatives, offer tunable stiffness, porosity, and degradation kinetics (Khan et al., 2024; Moulin et al., 1996). Electrospinning, in particular, yields fibrous meshes with high surface area and interconnected pores resembling native ECM (Keirouz et al., 2020; Law et al., 2017). Composite electrospun scaffolds also enable fine-tuning of mechanical performance by adjusting polymer ratios, with UV crosslinking enhancing fiber stability and reducing wettability (Keirouz et al., 2019).

Despite these advantages, synthetic polymers lack intrinsic biochemical cues and often require functionalization with adhesive peptides, such as Arginine-Glycine-Aspartic Acid (RGD) (Li, 2024) or coatings of proteins like collagen (Powell and Boyce, 2009) or laminin (Jiao and Cui, 2007) to promote cell attachment and differentiation. Hybrid strategies combining natural hydrogels with reinforcing scaffolds also enhance stability while preserving bioactivity. For example, fibrin gels reinforced with collagen–glycosaminoglycan matrices show marked increases in compressive and tensile modulus as well as resistance to contraction (Brougham et al., 2015), while bilayer constructs coupling electrospun membranes with hydrogel layers provide both dermal support and epidermal barrier properties (Franco et al., 2011).

#### 3.2.3 Decellularized dermal scaffolds

Decellularization, a widely applied strategy in regenerative medicine, has recently emerged as a method for generating physiologically relevant 3D skin models. By removing cellular components while preserving ECM and vascular structures, donor-derived dermis provides bioactive scaffolds that support adhesion, proliferation, and differentiation of skin-relevant cells. Although donor availability and processing remain limitations, these scaffolds are particularly attractive for applications requiring preserved vascular integrity, such as drug permeation and delivery studies (Zhang et al., 2022).

Protocols have evolved to improve ECM preservation. Enzymatic–mechanical approaches achieve complete decellularization while maintaining collagen, elastic fibers, glycosaminoglycans, vascular channels, and growth factor content (Bondioli et al., 2014). Detergent-free strategies, such as osmotic shock combined with cytoskeletal disruption, further retain ECM components and mechanical strength while supporting fibroblast infiltration (Farrokhi et al., 2018). More recently, decellularized dermis has been processed into hydrogels that, when recellularized with keratinocytes and fibroblasts, yield a stratified epidermis with enhanced differentiation, barrier function, and expression of markers such as transglutaminase 1 and keratin 14 (Sarmin and Connelly, 2022).

Beyond dermal reconstruction, decellularized matrices facilitate the integration of subcutaneous adipose tissue, thereby enabling the formation of trilayer constructs. In one example, keratinocytes and fibroblasts were seeded onto decellularized dermis and combined with mechanically processed human adipose tissue, which contained adipocytes, stromal cells, and microvascular elements. The resulting model maintained stable architecture and cellular viability for nearly a month (Workman et al., 2023), underscoring the potential of decellularized scaffolds for long-term, physiologically relevant skin equivalents.

### 3.3 Bioprinting techniques for skin fabrication

Bioprinting has emerged as a transformative technology for fabricating skin models, offering precise reconstruction of multicellular architecture and layered organization. This approach converges 3D printing, biomaterials science, and cell biology to create physiologically relevant skin equivalents through layer-by-layer deposition of hydrogel-based bioinks containing cells and bioactive molecules (Derman et al., 2025).

#### 3.3.1 Bioprinting modalities

Several bioprinting techniques have been explored to fabricate skin models, each with specific advantages and limitations: i) Extrusion-based bioprinting is the most widely used approach, employing pneumatic, piston, or screw-driven mechanisms for controlled bioink deposition. While enabling multi-material printing with spatial control over cell and ECM distribution, the inherent shear stress during extrusion can compromise cell viability and differentiation (Rossi et al., 2024); ii) Droplet-based bioprinting utilizes thermal or piezoelectric actuators; this method achieves high-resolution patterning with minimal cell damage. Thermal inkjet offers cost-effective precision, whereas piezoelectric variants better preserve cell viability. Both face challenges with nozzle clogging and limited biomaterial compatibility (Xu et al., 2025); iii) Laser-based bioprinting is a contact-free technique that uses focused laser pulses to generate microdroplets, enabling high spatial accuracy for complex tissue architectures. However, its technical complexity and high cost limit widespread adoption (Ventura, 2021); iv) Light-based bioprinting employs UV or visible light for photopolymerization, providing automated control and micron-scale resolution, particularly valuable for vascularized constructs. Challenges include potential cytotoxicity from photopolymerization and limitations in material selection (Zhang et al., 2023).

#### 3.3.2 Bioink development

Effective skin bioprinting requires bioinks that achieve a balance between printability, mechanical integrity, and biological functionality. Several materials have emerged as leading platforms, each offering distinct advantages. Among the most widely used, collagen-based bioinks provide a biomimetic ECM-like microenvironment that promotes keratinocyte adhesion, proliferation, and differentiation. Alternatively, decellularized ECM (dECM) bioinks, derived from skin or other tissues, retain native biochemical cues, including growth factors, glycosaminoglycans, and structural proteins, which enhance cell-matrix interactions and promote tissue-specific remodeling. For applications demanding high precision, gelatin methacrylate (GelMA) bioinks combine the biological recognition motifs of gelatin with tunable photocrosslinkable properties, enabling spatial control over mechanical stiffness and degradation kinetics. This makes GelMA suited for fabricating layered skin constructs with customized architectures. Furthermore, its versatility allows it to be blended with other biomaterials to be functionalized with bioactive molecules, thereby improving cellular behavior and tissue maturation post-printing (Derman et al., 2025).

#### 3.3.3 Transition from therapeutic to preclinical applications

Originally developed as advanced therapies for wound repair, bioprinted skin substitutes are now being repurposed as in vitro platforms for drug screening. These constructs often incorporate the key cellular components of native skin (keratinocytes for epidermal stratification and fibroblasts for dermal ECM). Additionally, they can also incorporate other cell types, like endothelial cells or their progenitors, to replicate vascular-like networks. Although these models do not support active perfusion or systemic absorption in vitro, maintaining them at the ALI and applying test compounds topically or basally enables the evaluation of keratinocyte proliferation, stratification, dermal remodeling, inflammation, cytotoxicity, and vascular responses. Studies by Dai et al. (2022) and Baltazar et al. (2020) have demonstrated how bioprinted skin models originally validated for implantation can be adapted for drug testing applications through optimization of culture duration and ALI maintenance protocols.

A significant advancement in this field was achieved by Rimal et al. (2021), who developed a vascularized human skin equivalent specifically designed for in vitro applications rather than clinical transplantation. This model employed a scaffold-free approach combined with dynamic bioreactor perfusion to create a full-thickness construct featuring ECM-coated fibroblasts, endothelial cells, and keratinocytes. Unlike conventional implant-oriented models, Rimal's system was engineered to maintain long-term vascular stability and tissue viability under physiologically relevant flow conditions. The dynamic perfusion system preserved ECM integrity by regulating angiogenic factors (VEGF, HIF1α) and protease activity (MMPs/TIMPs balance), while simultaneously enhancing epidermal barrier function and promoting the development of perfusable vascular networks. These engineered features proved valuable for preclinical applications, as evidenced by the model's improved performance in wound closure assays and its capability for real-time visualization of drug transport through vascular channels, a feature that significantly enhances its utility for pharmacokinetic studies.

### 3.4 Human pluripotent stem cell-based skin models

Skin organoids represent a distinct class of in vitro models that emerge through the self-organization of multiple cell types, typically derived from human pluripotent stem cells (hPSCs). These organoids recapitulate the features of native skin, including a stratified epidermis, dermal components, and, in some cases, skin appendages such as hair follicles and sebaceous glands (Hong et al., 2023; Sun et al., 2021). A defining feature of skin organoids is their capacity for spontaneous morphogenesis, often without externally imposed architectural templates (Ebner-Peking et al., 2021). While some protocols incorporate biomaterials like hydrogels to support cellular organization, the classification as an organoid depends on whether tissue architecture arises from intrinsic self-organization rather than manual assembly (Hong et al., 2023; Sun et al., 2021).

Because this review focuses on manually reconstructed 3D in vitro skin models, it falls outside the scope of organoids. Nevertheless, in vitro skin models may employ hPSC-derived cell types, particularly those generated from induced pluripotent stem cells (iPSCs). The use of hPSCs brings advantages over primary somatic cells, including reduced donor variability and the ability to generate patient-specific or genetically modified populations. hPSCs can be differentiated into a broad range of skin-resident cell types, such as keratinocytes, fibroblasts, endothelial cells and immune cells (Dinella et al., 2014), and incorporated into 3D skin constructs. Keratinocyte differentiation is characterized by the downregulation of pluripotency markers (e.g., octamer-binding transcription factor 4) and the upregulation of epidermal markers (e.g., keratin 5/keratin 14), which enables the formation of stratified epidermal layers comparable to those from primary cells (Khurana et al., 2021). Models may also involve genome editing, such as CRISPR/Cas9-mediated correction, or isogenic iPSCs, and are validated through morphological and molecular analysis (Khurana et al., 2021; Naito et al., 2019).

In addition to supporting the generation of healthy skin equivalents, hPSCs have enabled the development of disease-specific 3D skin models for rare genetic disorders, where primary patient-derived cells are scarce or lose phenotype over time. iPSCs derived from affected individuals retain disease-causing mutations and can be differentiated into relevant skin cell types, while isogenic controls generated via genome editing (e.g., CRISPR/Cas9) allow direct comparison within identical genetic backgrounds. For instance, iPSC-based 3D skin models of recessive dystrophic epidermolysis bullosa (Itoh et al., 2011) and systemic sclerosis (Kim et al., 2018) have successfully recapitulated key pathological features, validating their utility for preclinical research and therapeutic screening.

## 4 Wound healing: acute progression vs. chronic impairment

### 4.1 Phases of acute wound healing

**Fig. 3:**
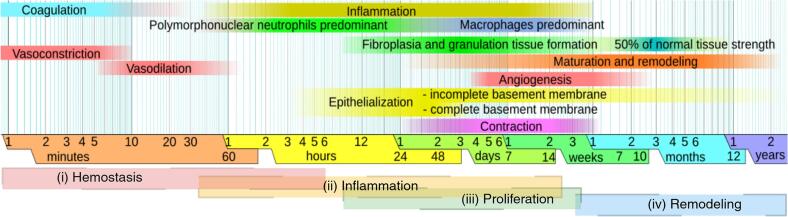
Temporal organization of the acute wound healing process and its physiological events. (i) Hemostasis begins immediately after tissue injury and is marked by vasoconstriction, platelet aggregation, and fibrin clot formation. (ii) The inflammatory phase (from ∼several hours to 14 days) is characterized by the sequential infiltration of neutrophils and macrophages. Neutrophils mediate pathogen clearance via reactive oxygen species and neutrophil extracellular traps (NETs), whereas macrophages transition from a pro-inflammatory (M1) to a reparative (M2) phenotype to coordinate inflammation resolution, ECM remodeling, and angiogenesis (Cioce et al., 2024; Willenborg and Eming, 2014). (iii) During the proliferative phase (from ∼day 1 to 21 days), keratinocytes, fibroblasts, endothelial cells, and various immune subsets cooperate to rebuild tissue. Fibroblasts proliferate and differentiate into myofibroblasts, contributing to ECM deposition and wound contraction. Epidermal stem cells, assisted by dermal fibroblasts and mesenchymal stromal cells, drive re-epithelialization. (iv) The remodeling phase (from ∼day 21 and extending up to 1 year) involves ECM maturation and reorganization of collagen fibers under the regulation of fibroblasts and macrophages. Matrix metalloproteinases (MMP-1, MMP-9) and their inhibitors (TIMPs) regulate collagen turnover, contributing to tensile strength recovery and tissue homeostasis (Fernández-Guarino et al., 2023; Rodrigues et al., 2019). This timeline represents the events during uncomplicated healing, but deviations from this sequence underlie chronic wound pathogenesis. Figure adapted from Häggström, M. Public domain (Häggström, 2023).

Wound healing is a biological process that restores skin integrity following injuries such as abrasions, burns, and surgical incisions. The repair of damaged skin relies on a highly coordinated process that involves four overlapping phases: hemostasis, inflammation, proliferation, and remodeling. In acute wounds, this process typically unfolds over specific timeframes: hemostasis occurs within hours, inflammation lasts up to 14 days, proliferation takes place from day 1 to around day 21, and remodeling continues from 21 days to 1 year (Fig. 3). These intervals represent the general trajectory of physiological healing, assuming the absence of complications (Reinke and Sorg, 2012).

The hemostasis phase represents the immediate response to tissue injury and is initiated by platelet activation, which promotes the release of growth factors, including PDGF, TGF-β, and VEGF. These factors stimulate fibrin clot formation, which not only prevents further blood loss but also provides a provisional matrix for immune cell infiltration (Fernández-Guarino et al., 2023).

Next, the inflammatory phase is characterized by the recruitment and activation of immune cells at the wound site, where they eliminate pathogens and initiate the repair process. Neutrophils are the first to infiltrate the wound, clearing pathogens through phagocytosis, an oxidative burst, and the formation of neutrophil extracellular traps (NETs). While essential for defense, their persistence can lead to chronic inflammation (Cioce et al., 2024). Soon after, monocytes differentiate into macrophages. Initially adopting a pro-inflammatory M1 phenotype, macrophages secrete cytokines such as TNF-α, IL-1β, and IL-6 to enhance their defense mechanisms. As healing progresses, they shift to a reparative M2 phenotype, promoting resolution of inflammation, ECM synthesis, and angiogenesis through factors such as IL-10, TGF-β, and VEGF-A (Willenborg and Eming, 2014). Other immune cells also contribute. Mast cells release histamine, tryptase, and cytokines to modulate vascular permeability and fibroblast activity. Langerhans cells act as resident sentinels, capturing antigens and migrating to draining lymph nodes to orchestrate adaptive immune responses, while locally producing cytokines and chemokines that recruit leukocytes and modulate keratinocyte activity (Neagu et al., 2022a). T lymphocyte subsets further fine-tune the immune response: Th1 cells sustain inflammation via IFN-γ; Th2 cells support tissue repair and fibrosis through IL-4 and IL-13; Th17 and Th22 cells release IL-22 to stimulate re-epithelialization and angiogenesis; and regulatory T cells suppress inflammation and promote M2 polarization. Additional contributors include γδ T cells, which release IGF-1 and IL-17 to enhance keratinocyte survival and leukocyte recruitment, as well as dendritic cells, which modulate T cell responses and may influence proliferation and granulation tissue formation (Cioce et al., 2024; Willenborg and Eming, 2014).

During the proliferative phase, fibroblasts expand and differentiate into myofibroblasts, contributing to ECM production and wound contraction. Angiogenesis is stimulated by hypoxia and growth factors, such as VEGF and PDGF, which involve endothelial cells, pericytes, and M2-like macrophages. Endothelial cells form new capillary sprouts through the dynamics of tip and stalk cells, which are regulated by Notch signaling, while circulating progenitors aid in vessel formation. Simultaneously, keratinocytes migrate and proliferate to rebuild the epidermis, supported by dermal fibroblasts and subcutaneous mesenchymal stem cells. Epidermal stem cells, initially unipotent, acquire plasticity after injury to restore the skin's layers. Langerhans cells and dermal dendritic cells maintain immune surveillance, and epidermal γδ T cells produce growth factors that activate keratinocyte proliferation. In the final remodeling phase, fibroblasts and macrophages coordinate ECM reorganization. Collagen fibers are realigned and cross-linked to reestablish tissue structure and tensile strength (Fernández-Guarino et al., 2023; Rodrigues et al., 2019).

### 4.2 Pathophysiology of chronic wounds

In contrast to the normal, temporarily organized healing process, chronic wounds fail to progress through these sequential phases effectively. The non-healing wounds include diabetic ulcers (DUs), also referred to as diabetic foot ulcers (DFUs), venous ulcers (VUs), non-healing pressure ulcers (NHPUs), and arterial insufficiency ulcers (AIUs) (Federman et al., 2016; Gould et al., 2016; Jung et al., 2016; Marston et al., 2016).

The development of each chronic wound type results from distinct pathophysiological conditions, influenced by systemic factors, such as patient age, malnutrition, medication use (e.g., corticosteroids and immunosuppressants), obesity, and underlying medical conditions (e.g., cardiac failure, vascular disease, diabetes mellitus, hyperlipidemia, hypertension, autoimmune disorders), as well as local factors, such as vascular deficits (e.g., venous or arterial insufficiency), neuropathy, infections, and mechanical stress (e.g., localized pressure) (Tiwari and Pathak, 2023).

The DFUs arise from a pathogenic triad of neuropathy, angiopathy, and immunopathy. Peripheral sensory neuropathy leads to trauma and foot deformities, while motor and autonomic dysfunction cause abnormal pressure distribution and skin dryness. Concurrent micro- and macroangiopathy impair perfusion and oxygen delivery, and hyperglycemia further disrupts immune cell function, creating a milieu prone to tissue breakdown and impaired repair (Ellis et al., 2018; Fernández-Guarino et al., 2023; Parveen et al., 2025). The VUs, in contrast, are primarily caused by chronic venous hypertension due to valvular incompetence or obstruction. This increased pressure causes extravasation of fibrinogen, erythrocytes, and inflammatory mediators into the dermis, leading to perivascular fibrin cuff formation, hemosiderin deposition, and a sustained pro-inflammatory state. These changes impede oxygen and nutrient diffusion, resulting in tissue hypoxia and ulceration, typically in the gaiter area of the leg (Alavi et al., 2016; Ellis et al., 2018; Marston et al., 2016).

The NHPUs follow a different mechanistic pathway, resulting from sustained, unrelieved mechanical pressure and shear forces that exceed capillary closing pressure (Gould et al., 2016; Jung et al., 2016). This causes direct ischemic necrosis of the skin and underlying tissues, with damage often beginning at the deeper muscle-bone interface. Cycles of ischemia and reperfusion further exacerbate injury through oxidative stress and inflammation (Ellis et al., 2018; Fernández-Guarino et al., 2023). Finally, the AIUs are a consequence of inadequate tissue perfusion caused by peripheral arterial disease. Atherosclerotic occlusion leads to limb ischemia, and the resulting tissue hypoxia and malnutrition render distal extremities, vulnerable to minor trauma. This leads to painful, necrotic, “punched-out” wounds that lack the perfusion necessary for healing (Federman et al., 2016).

**Fig. 4:**
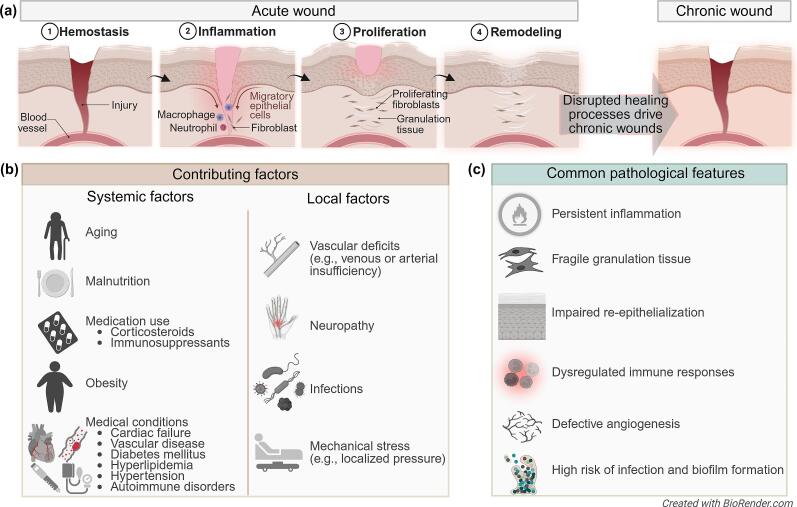
Pathophysiology of chronic non-healing wounds: comparison with physiological healing, contributing factors, and pathological hallmarks. (a) Acute wound healing progresses through four temporally regulated and overlapping phases: hemostasis, inflammation, proliferation, and remodeling. In contrast, chronic wounds exhibit disruption of this orderly sequence, resulting in persistent tissue damage and impaired healing. (b) Chronic wounds arise from interactions between systemic and local factors. Systemic factors include aging, malnutrition, use of corticosteroids or immunosuppressants, obesity, and underlying conditions such as cardiac failure, vascular disease, diabetes mellitus, hyperlipidemia, hypertension, and autoimmune disorders. Local factors comprise vascular deficits (e.g., venous or arterial insufficiency), neuropathy, infections, and mechanical stress (e.g., prolonged pressure). (c) Despite their different etiologies, chronic wounds share several common pathological features: persistent inflammation, fragile and non-functional granulation tissue, impaired re-epithelialization, dysregulated immune responses, defective angiogenesis, and increased susceptibility to infection and biofilm formation. Created in https://BioRender.com.

Additionally, infection, particularly when associated with biofilm formation, acts as an aggravating factor across all chronic wound types. Microbial biofilms perpetuate a state of persistent inflammation, directly damage host tissue through bacterial enzymes and toxins, and shield bacteria from both the immune system and antimicrobial agents, arresting the healing process (Zhao et al., 2013). Despite their differences, these wound types share several common hallmarks: persistent inflammation, fragile granulation tissue, impaired re-epithelialization, dysregulated immune responses, defective angiogenesis, and a high risk of infection and biofilm formation (Fig. 4) (Fernández-Guarino et al., 2023; Hofmann et al., 2023a).

## 5 3D models of acute and chronic skin wounds

**Fig. 5:**
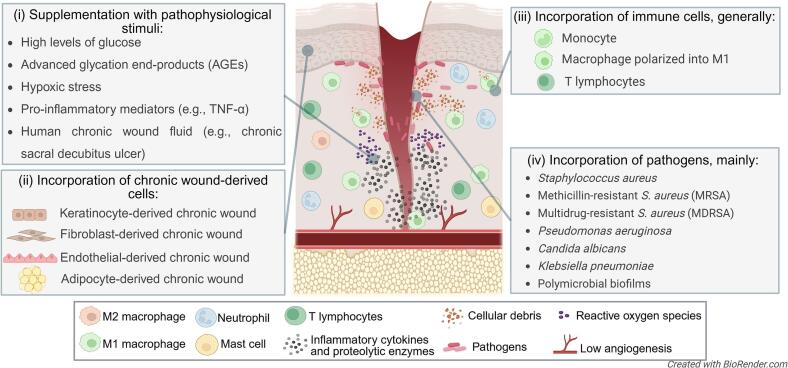
Strategies for mimicking the chronic wound microenvironment in 3D in vitro skin models. Main components incorporated into engineered models to recapitulate the complex microenvironment of chronic, non-healing wounds. (i) Supplementation with pathophysiological stimuli, such as high glucose levels, advanced glycation end-products (AGEs), hypoxic stress, pro-inflammatory cytokines (e.g., TNF-α), and chronic wound fluid (e.g., from sacral decubitus ulcers), helps to reproduce the biochemical conditions of the chronic wound bed. (ii) Chronic wound-derived cells include keratinocytes, fibroblasts, endothelial cells, and adipocytes obtained directly from patient wound tissues, preserving disease-specific features such as impaired proliferation, aberrant signaling, and altered matrix remodeling. (iii) Immune cell incorporation, particularly of monocytes, macrophages polarized into pro-inflammatory M1 phenotypes, and T lymphocytes, enables modeling of the persistent inflammation typical of chronic wounds. (iv) Pathogen integration replicates wound colonization with clinically relevant species, including *Staphylococcus aureus*, methicillin-resistant *S. aureus* (MRSA), multidrug-resistant *S. aureus* (MDRSA), *Pseudomonas aeruginosa*, *Candida albicans*, *Klebsiella pneumoniae,* and polymicrobial biofilms. The central illustration depicts hallmarks of chronic wounds, including cellular debris accumulation, elevated reactive oxygen species (ROS), excessive inflammatory cytokine and protease activity, impaired angiogenesis, and sustained immune infiltration, all of which contribute to defective wound healing. Created in https://BioRender.com.

Strategies to model skin injury in vitro range from basic wound induction (section 5.1) through increasingly complex pathophysiological elements (5.2, 5.3, 5.4, 5.5, 5.6) that incorporate the pathophysiological hallmarks of chronic wounds. Fig. 5 summarizes key approaches to recreating the chronic wound microenvironment, while Table 1 provides examples of human 3D skin models, detailing cell sources, dermal architecture, wound induction methods, and validation endpoints. It is worth mentioning that while numerous models have been validated through structural, functional, and molecular analyses, most rely on internal characterization rather than direct benchmarking against clinical or in vivo data. For mechanistic research, such validation may be sufficient. However, for models intended as alternatives to animal testing, establishing correlation with clinical outcomes or animal model data becomes imperative to ensure predictive value.

**Table 1:** Human skin equivalents (HSE) employed to model wound healing and chronic wound pathophysiology: cell sources, dermal and subcutaneous tissue composition, wound induction methods, and validation strategies. The models are grouped according to their structural complexity.

Model type	Cell Type	Dermal and subcutaneous tissue composition	Method of wound induction	Method for mimicking a chronic wound milieu	Model validation	Ref.
**HSEs without subcutaneous tissue, immune, or vascular components**						
- HSE; - No subcutaneous tissue, immune cells, or vascular components included.	- Normal human keratinocytes; - Normal human dermal fibroblasts.	- Dermal layer: type I collagen hydrogel populated with fibroblasts.	- Incisional wounds (using scalpel) or excisional wounds (using biopsy punch); - The wounded tissue is transferred onto a second, freshly contracted type I collagen gel to support reepithelialization.	- Not specific to a chronic wound; - Use of phenotypically modified fibroblasts: e.g., fibroblasts cultured on denatured collagen films or long-term passaged fibroblasts to emulate altered stromal signaling and delayed repair.	- Histology (H&E) to assess re-epithelialization and tissue morphology; - Immunohistochemical analysis; - Phenotypic response of epithelium (e.g., keratinocyte proliferation, migration, and differentiation).	Egles et al. (2010)
- HSE; - No subcutaneous tissue, immune cells, or vascular components included.	- Normal human keratinocytes; - Normal human dermal fibroblasts.	- Dermal layer: three-layered construct composed exclusively of fibroblast-derived dermal sheets; - Two fibroblast sheets were stacked and cultured with ascorbic acid to produce ECM-rich layers; - After wounding, a third fibroblast sheet was placed underneath to serve as the wound bed.	- Excisional wounds (using biopsy punch); - The wounded tissue is transferred onto a third fibroblast sheet to support reepithelialization.	- Not specific to a chronic wound; - Modulation of the wound environment was achieved by applying fibrin or platelet-rich plasma, which accelerated reepithelialization but delayed DEJ reformation.	- Histology (H&E) to assess re-epithelialization and tissue morphology; - Expression patterns of differentiation markers (K1/K10, filaggrin, loricrin); - DEJ reformation assessed via laminin 5 and collagen IV deposition; - Keratinocyte proliferation by Ki67 index; - Quantification of wound closure via digital image analysis.	Laplante et al. (2001)
- HSE; - No subcutaneous tissue, immune cells, or vascular components included.	- Normal human keratinocytes; - Normal human dermal fibroblasts.	- Dermal layer: collagen type I hydrogel embedded with human dermal fibroblasts.	- Excisional wounds (using nitrogen-cooled metal rod) or superficial wounds using scalpel incision; - The wounded tissue is transferred onto a second fibroblast-containing collagen matrix to support reepithelialization.	- Not specific to a chronic wound.	- Histology (H&E) and immunohistochemical to assess reepithelialization in superficial and full-thickness wounds; - Expression of keratinocyte differentiation markers (K10, K16, K17, involucrin, and SKALP); - DEJ regeneration evaluated via laminin 5, collagen IV/VII, and integrin α6/β4 deposition; - Differential DJ-1 expression identified by 2D-PAGE and validated by immunostaining.	El Ghalbzouri et al. (2004)
- HSE adapted from RealSkin®; - No subcutaneous tissue, immune cells, or vascular components included.	- Normal human keratinocytes; - Normal human dermal fibroblasts already embedded into RealSkin® dermal equivalent.	- Dermal layer: Dermal matrix (lattice RealSkin®) - Construct supported by a metal ring to spatially restrict initial seeding.	- Cell-free central area created by metal ring barrier; - Ring removed after 10 days to allow centripetal migration of keratinocytes, mimicking re-epithelialization phase.	- Not specific to a chronic wound.	- Reepithelialization evaluated by histological analysis of migration area, and epidermal thickness; - Quantification of keratinocyte migration kinetics; - Evaluation of pro-healing agents (punicic acid, ellagic acid, ascorbic acid) on epithelialization speed.	Deshayes et al. (2018)
- HSE; - No subcutaneous tissue, immune cells, or vascular components included.	- HaCaT keratinocytes - Normal human dermal fibroblasts.	- Dermal layer: Type I collagen hydrogel embedded with fibroblasts.	- Excisional wounds (using biopsy punch).	- Chronic wound fluid (CWF, 10%) collected from sacral pressure ulcers (≥ 6 weeks duration) was added to the culture medium; - Acute wound fluid (AWF; 10%) condition was also assessed.	- Histological (H&E) analysis of wound bed and margins; - Immunohistochemistry for Ki67 (proliferation), CXCR4 (migration), and FAP (active fibroblasts); - Spatial analysis of keratinocyte distribution and proliferation at the wound margins and central wound area (to assess re-epithelialization dynamics and lateral cell migration under acute or chronic wound fluid stimulation).	Besser et al. (2017)
- HSE; - No subcutaneous tissue, immune cells, or vascular components included.	- HaCaT keratinocytes - Normal human dermal fibroblasts.	- Dermal layer: Type I collagen hydrogel embedded with fibroblasts.	- Excisional wounds (using biopsy punch).	- Chronic wound fluid (CWF, 10%) collected from sacral pressure ulcers (≥6 weeks duration) was added to the culture medium; - Acute wound fluid (AWF; 10%) condition was also assessed.	- Histological (H&E) analysis of wound bed and margins; - Immunohistochemistry for Ki67 (proliferation), CXCR4 (migration), and Caspase 3 (apoptosis); - Hyperspectral imaging to monitor spectral reflectance signatures over time; - Correlation of reflectance clusters with cellular density.	Wahabzada et al. (2017)
- HSE; - No subcutaneous tissue, immune cells, or vascular components included.	- Normal human keratinocytes; - Primary human dermal fibroblasts isolated from healthy non-diabetic foot skin, non-ulcerated diabetic foot skin, or diabetic foot ulcer tissue.	- Dermal layer: type I collagen hydrogel populated with fibroblasts.	- Excisional wounds (using biopsy punch).	- Use of patient-derived fibroblasts from diabetic foot ulcer tissue.	- Histological (H&E) evaluation of epidermal stratification and wound closure; - BrdU staining for keratinocyte proliferation; - Quantification of re-epithelialization in wounded HSE; - ECM thickness measurements;	Maione et al. (2015)

**HSEs with subcutaneous tissue**						
- HSE; - Subcutaneous tissue included; - No immune cells, or vascular components included.	- Normal human keratinocytes; - Normal human dermal fibroblasts; - Human adipocytes differentiated from mesenchymal stem cells (hMSCs).	- Dermal layer: collagen type I hydrogel embedded with fibroblasts (bioprinted concentric pattern); - Subcutaneous layer: collagen type I matrix seeded with adipocyte-differentiated hMSCs; - All layers sequentially bioprinted.	- No wound applied.	- Not specific to a chronic wound.	- Histological and SEM analysis of full-thickness architecture; - Immunofluorescence for differentiation markers (K1, K10, K15, involucrin); - Bodipy staining and qPCR for adipogenic markers (PPARγ, PPARβ, PDK4); - TEER measurements for barrier integrity; - ATP assay for viability; - RNA-seq and GO enrichment analysis (1081 DEGs: ECM remodeling, lipid metabolism, keratinocyte differentiation).	Avelino et al. (2024)

**HSEs with vascular components**						
- HSE with synthetic vascular scaffold; - Vascular component included; - No subcutaneous tissue or immune cells included.	- Normal human keratinocytes; - Normal human dermal fibroblasts; - Human dermal microvascular endothelial cells (HDMECs).	- Dermal layer: de-epidermized dermis seeded with fibroblasts; - Vascular layer: electrospun PHBV scaffold with perfusable hollow channels populated with HDMECs.	- No wound applied.	- Not specific to a chronic wound.	- Histological (H&E) and immunohistochemical (CD31) analysis of endothelial cell outgrowth; - Quantification of angiogenic parameters (tube length, branch points) via ImageJ (Angiogenesis Analyzer plugin, AngioTool); - *Ex ovo* CAM assay for in vivo validation of vascularization; - Biomechanical characterization of PHBV scaffold (tensile and suture retention tests); - SEM for morphology of PHBV scaffold.	Dikici et al. (2020)
- HSE, bioreactor-cultivated; - Vascular component included; - No subcutaneous tissue or immune cells included.	- Normal human keratinocytes; - Normal human dermal fibroblasts; - HUVECs.	- Dermal layer: fibroblasts + HUVECs coated with a nano-film of ECM using layer-by-layer deposition of fibronectin and gelatin.	- Laser ablation.	- Not specific to a chronic wound; - Acute wound used to validate vascularized HSE system.	- Histological (H&E), confocal and electron microscopy analyses; - RT-PCR for angiogenesis, ECM and inflammation markers (VEGFA, HIF1A, MMPs, TIMPs); - Barrier function via TEER; - ECM protein expression (e.g., fibronectin, filaggrin); - Quantification of wound closure (static vs. flow).	Rimal et al. (2021)
- HSE; - Vascular component included; - No subcutaneous tissue or immune cells included.	- Human diabetic keratinocytes (from type 2 diabetic donors); - Human diabetic dermal fibroblasts (from type 2 diabetic donors); - Diabetic HUVECs (from type 2 diabetic donors).	- Dermal layer: Photocrosslinked GelMA hydrogel containing atelocollagen-coated fibroblasts and HUVECs.	- Excisional wounds (using biopsy punch); wound filled with therapeutic hydrogel (atelocollagen, sodium alginate, ascorbic acid).	- Use of primary cells from type 2 diabetic patients: keratinocytes, fibroblasts, and HUVECs; - Culture in high glucose medium (25 mM).	- Immunofluorescence (K5 for epidermal stratification; vimentin for dermal fibroblasts; CD31 for endothelial cells); - Masson's trichrome staining (for ECM distribution); - Assessment of fibroblast and keratinocyte migration into wound.	Ozdogan et al. (2021)

**HSEs with immune cells**						
- HSE; - Immune cells included; - No subcutaneous tissue or vascular components included.	- Normal human keratinocytes; - Human foreskin fibroblasts, adult nondiabetic foot fibroblasts, or diabetic foot ulcer fibroblasts; - Peripheral blood-derived monocytes (differentiated into macrophages).	- Dermal layer: endogenous human ECM produced by fibroblasts cultured with ascorbic acid over 3 weeks.	- Excisional wounds (using biopsy punch).	- Use of patient-derived fibroblasts from diabetic foot ulcer tissue; - Incorporation of monocyte-derived macrophages.	- Histology (H&E) to assess re-epithelialization and tissue morphology; - Immunohistochemistry for Keratin 10 (differentiation), Ki67 (proliferation), CD163 (macrophages), Collagen I/III; - Picrosirius red staining for collagen fiber orientation; - Comparison of healing dynamics using healthy fibroblasts vs. diabetic foot ulcer fibroblasts.	Smith et al. (2020)
- HSE; - Immune cells included; - No subcutaneous tissue or vascular components included.	- Normal human keratinocytes; - Adult nondiabetic foot fibroblasts, or diabetic foot ulcer fibroblasts; - Peripheral blood-derived monocytes (from diabetic or control patients), differentiated into macrophages.	- Dermal layer: Type I bovine collagen matrix populated with fibroblasts and macrophages (polarized or unpolarized).	- No wound applied.	- Use of patient-derived fibroblasts from diabetic foot ulcer tissue; - Incorporation of monocytes from diabetic patients to simulate persistent M1 macrophage phenotype.	- Histological analysis (H&E); - Immunofluorescence for K10 (keratinocyte differentiation), CD68 (macrophages), HLADR, CD163, CD206 (M1/M2 markers); - ELISA and multiplex cytokine assays for IL-1β, IL-6, IL-8, IL-10, IL-13, IL-2, TNF-α; - ImageJ quantification of M1/M2 macrophage markers.	Smith et al. (2021)
- HSE; - Immune cells included; - No subcutaneous tissue or vascular components included.	- Normal human keratinocytes; - Normal human dermal fibroblasts; - Human peripheral blood monocytes; - Human peripheral blood T cells (CD3+).	- Dermal layer: MatriDerm® collagen-elastin matrix seeded with fibroblasts; - Immune cells added post-construction to dermal side or between transwell and dermis.	- Thermal burn injury using a copper plate (80–90°C, 20s contact).	- Not specific to a chronic wound; - Inclusion of immune components (monocytes or T cells) post-burn to mimic innate or adaptive immune response to burn injury.	- Histological (H&E) and LDH staining of burn injury and viability; - Immunohistochemistry for CD68 (macrophages), CD3 (T cells); - Flow cytometry for phenotype of macrophages (CD68, HLA-DR, CD14, CD11b, CD163) and T cells (CD4, CD25, CD127, CXCR3, CCR4, CCR6); - Cytokine multiplex assay (e.g., IL-6, IL-8, IFN-γ, IL-10, IL-12p70, IP-10, TGF-β1).	Mulder et al. (2023)

**HSEs with multiple advanced components**						
- HSE; - Subcutaneous tissue included; - Vascular component included; - No immune cells included	- Normal human keratinocytes; - Human diabetic dermal fibroblasts; - Human diabetic preadipocytes (isolated from type II donors); - HUVECs.	- Dermal layer: skin-derived decellularized ECM bioink containing diabetic fibroblasts; - Subcutaneous layer: adipose-derived decellularized ECM bioink containing differentiated diabetic preadipocytes; - Vascular layer: coaxially printed vascular channels using vascular-derived decellularized ECM and sodium alginate bioink encapsulating HUVECs, placed in the subcutaneous layers; - All layers constructed using a 3D bioprinting system.	- Mechanical wounding using 16-gauge needle during epidermal differentiation.	- Use of diabetic patient-derived cells (fibroblasts and preadipocytes); - High-glucose (30 mM) culture medium; - Inclusion of vascular and adipose compartments to simulate diabetic skin microenvironment with vascular dysfunction and insulin resistance.	- Histology (H&E) of epidermal thickness and reepithelialization; - Immunostaining for Ki67, K10, involucrin, phospho-IR, collagen I, ZO-1, CD31; - Lipid accumulation via Bodipy staining; - Gene expression analysis by qRT-PCR (IL-6, IL-8, MCP-1, TNF-α, K10, IR); - ELISA for IL-6 and TNF-α in wound-conditioned media; - ROS measurement using ROS/RNS assay; - Glucose uptake assay with 2DG and insulin perfusion; - Vascular functionality via perfusion and FITC-dextran permeability; - Fibroblast contractility and endothelial sprouting assays.	Kim et al. (2021)
- HSE; - Subcutaneous tissue included; - Immune cells and neurons included.	- Normal human keratinocytes; - Normal human dermal fibroblasts; - Human induced neural stem cells (hiNSCs); - Lipoaspirate containing 62% mature adipocytes and preadipocytes, 27.4% immune cells (17.4% macrophages and 6.2% T lymphocytes), 5.7% endothelial cells, and 4.5% smooth muscle cells (Abbott et al., 2016).	- Dermal layer: crosslinked silk-collagen hydrogel populated with fibroblasts; - Subcutaneous layer: porous silk sponge with human lipoaspirate (containing adipocytes, preadipocytes, endothelial cells, smooth muscle pericytes, and macrophages); - Neuronal component: hiNSC-containing collagen gel interfaced with subcutaneous layer.	- No wound applied.	- Not specific to a chronic wound; - Model developed for long-term immune and neural interaction studies.	- Histology (H&E); - Immunohistochemical (βIII-Tubulin for neurons, CD68 for macrophages); - qRT-PCR for macrophage and inflammatory markers (CD68, CSF1, IL-6, RANTES, NOS2, ACRP30); - Vybrant™ DiD cell tracer (to track hiNSCs within hypodermal compartment); - Cytokine array: IL-6, IL-8, MCP-1, MCSF-1, GRO; - Proteomic analysis via LC-MS/MS; - Glycerol secretion for adipose functionality; - Mechanical evaluation (durometer and compression tests).	Vidal et al. (2019)

**Models incorporating pathogens or biofilms**						
- HSE (Labskin®); - No subcutaneous tissue, immune cells, or vascular components included.	- Commercially available Labskin® (contains primary human keratinocytes and fibroblasts).	- Pre-fabricated Labskin® model comprising a stratified epidermis and dermal fibroblast-populated matrix.	- Incisional wound using scalpel blade.	- Not specific to a chronic wound; - Inoculation with: *S. aureus* NCTC13435 and MSSA476, *K. pneumoniae* KP257, and *P. aeruginosa* PS1054.	- Mass spectrometry-based proteomic profiling (LESA-MS) of infected and control samples; - Top-down MS/MS analysis using mass spectrometers; - Identification of bacterial and host proteins via ProSight software; - Comparison across infected, wounded, and control conditions.	Havlikova et al. (2020)
- HSE; - No subcutaneous tissue, immune cells, or vascular components included.	- Normal human keratinocytes; - Normal human dermal fibroblasts.	- Dermal layer: rat-tail collagen hydrogel populated with fibroblasts.	- Thermal wound using blunt metal bar cooled in liquid nitrogen and applied for 15 s without pressure.	- Not specific to a chronic wound; - Inoculation with: methicillin-resistant *Staphylococcus aureus* (MRSA, strain LUH14616).	- Histological (H&E) and PAS/Alcian blue staining; - Immunohistochemistry for keratins (K10, K16, K17), Ki67 (proliferation), TLR2, collagen IV, hBD-2, hBD-3, LL-37; - ELISA for cytokines (IL-4, IL-6, IL-8, IL-10) and antimicrobial peptides (hBD-2, hBD-3); - qPCR for gene expression analysis (e.g., hBDs, cytokines); - Quantification of adherent/detachable MRSA colonies via CFU assays.	Haisma et al. (2013)
- HSE; - No subcutaneous tissue, immune cells, or vascular components included.	- Normal human keratinocytes; - Normal human dermal fibroblasts.	- Dermal layer: decellularized human dermis populated with fibroblasts.	- Thermal injury using heated metal rod for 6 s.	- Not specific to a chronic wound; - Inoculation with: clinical isolates of *Pseudomonas aeruginosa* (SOM1) or *Staphylococcus aureus* (S-235), and lab strain *S. aureus* NCTC 6571.	- Histological (H&E) and Gram staining; - Quantification of bacterial colonization by CFU assay from homogenized constructs; - Immunofluorescence for pancytokeratin (epidermal integrity) and type IV collagen (basement membrane).	Shepherd et al. (2009)
- HSE; - No subcutaneous tissue, immune cells, or vascular components included.	- Normal human keratinocytes; - Normal human dermal fibroblasts.	- Dermal layer: fibrin matrix containing embedded fibroblasts.	- No wound applied.	- Not specific to a chronic wound; - Inoculation with: *Staphylococcus aureus* SH1000, *Staphylococcus epidermidis*, *Propionibacterium acnes*, and *Malassezia furfur*	- Histological (H&E); - Immunohistochemistry for markers of epidermal differentiation (keratins 1, 6, 10, 16; filaggrin) and basement membrane (collagen IV); - Barrier integrity assessed by TEWL and surface pH measurements; - Bacterial colonization quantified by CFU; - Use of *S. aureus* lux strain to correlate luminescence with viable counts.	Holland et al. (2008)
- HSE; - No subcutaneous tissue, immune cells, or vascular components included.	- Normal human keratinocytes; - Normal human dermal fibroblasts.	- Dermal layer: fibrin matrix containing embedded fibroblasts.	- No wound applied.	- Not specific to a chronic wound; - Inoculation with: *Staphylococcus epidermidis* or *Staphylococcus aureus.*	- Bacterial colonization quantified by CFU; - RNA stabilization and isolation for microarray-based differential gene expression analysis; - Gene expression profiling with Agilent whole-genome oligo microarrays and LUMINATOR software.	Holland et al. (2009)
- RHE (EpiSkin®); - No subcutaneous tissue, immune cells, or vascular components included.	- Commercially available EpiSkin® (contains primary human keratinocytes).	- Not applicable (only epidermal component).	- No wound applied.	- Not specific to a chronic wound; - Biofilm layered atop intact RHE comprising eleven aerobic, anaerobic, and fungal species (including *Candida albicans, Staphylococcus aureus*, and *Pseudomonas aeruginosa*)	- Histological (H&E); - qPCR gene profiling of inflammatory markers (e.g., IL-6, IL-8, TNF-α); - Proteomic profiling via Olink platform (92 inflammation biomarkers); - ELISA confirmation for IL-8, VEGFA, IL-18.	Brown et al. (2022)
- HSE (Apligraf®); - No subcutaneous tissue, immune cells, or vascular components included.	- Commercially available Apligraf® (contains primary human keratinocytes and fibroblasts).	- Pre-fabricated Apligraf® model comprising a stratified epidermis and dermal fibroblast-populated matrix.	- Excisional wounds (using biopsy punch).	- Not specific to a chronic wound; - Inoculation with: *P. aeruginosa* or *S. aureus.*	- Histological (H&E); - Epifluorescence microscopy (Calcofluor white for polysaccharide biofilm matrix; Ethidium bromide for microbial DNA visualization).	Charles et al. (2009)

2DG: 2-Deoxy-d-Glucose; 2D-PAGE: Two-dimensional polyacrylamide gel electrophoresis; ACRP30: Adipocyte complement-related protein of 30 kDa; BODIPY: Boron-dipyrromethene; CAM: Chorioallantoic membrane; CCR4: C—C Chemokine receptor type 4; CCR6: C—C Chemokine receptor type 6; CD11b: Cluster of differentiation 11b; CD127: Cluster of differentiation 127; CD14: Cluster of differentiation 14; CD163: Cluster of differentiation 163; CD206: Cluster of differentiation 206; CD25: Cluster of differentiation 25; CD3: Cluster of differentiation 3; CD31: Cluster of differentiation 31; CD4: Cluster of differentiation 4; CD68: Cluster of differentiation 68; CFU: Colony-forming unit; CSF1: Colony stimulating factor 1; CXCR3: C-X-C Chemokine receptor type 3; CXCR4: C-X-C chemokine receptor type 4; DEGs: Differentially Expressed Genes; DEJ: Dermoepidermal junction; DJ-1: Protein DJ-1; ECM: Extracellular matrix; ELISA: Enzyme-Linked Immunosorbent Assay; FAP: Fibroblast activation protein alpha; FITC: Fluorescein Isothiocyanate; GO: Gene Ontology; GRO: Growth-Regulated Oncogene; HaCat: Human immortalized keratinocytes; hBD-2: Human Beta-Defensin 2; hBD-3: Human Beta-Defensin 3; HDMECs: Human dermal microvascular endothelial cells; HIF1A: Hypoxia-Inducible Factor 1 Alpha; hiNSCs: Human induced neural stem cells; HLA-DR: Human Leukocyte Antigen – DR isotype; HSE: Human Skin Equivalent; HUVECs: Human umbilical vein endothelial cells; IFN-γ: Interferon-gamma; IL-10: Interleukin-10; IL-12p70: Interleukin-12 heterodimer; IL-13: Interleukin-13; IL-1β: Interleukin-1 beta; IL-2: Interleukin-2; IL-4: Interleukin 4; IL-6: Interleukin-6; IL-8: Interleukin-8; IP-10: Interferon gamma-induced protein 10; IR: Insulin Receptor; K1, K10, K15, K16, K17: Keratin 1, Keratin 10, Keratin 15, Keratin 16, Keratin 17; LDH: Lactate Dehydrogenase; LL-37: Human cathelicidin antimicrobial peptide LL-37; MCP-1: Monocyte Chemoattractant Protein-1; MCSF-1: Macrophage Colony-Stimulating Factor 1; MMPs: Matrix Metalloproteinases; MRSA: Methicillin-Resistant *Staphylococcus aureus*; NOS2: Nitric Oxide Synthase 2; PDK4: Pyruvate Dehydrogenase Kinase Isoform 4; PHBV: poly-3-hydroxybutyrate-*co*-3 hydroxyvalerate; Phospho-IR: Phosphorylated Insulin Receptor; PPARβ: Peroxisome Proliferator-Activated Receptor Beta; PPARγ: Peroxisome Proliferator-Activated Receptor Gamma; qPCR: Quantitative Polymerase Chain Reaction; RANTES: Regulated upon Activation, Normal T cell Expressed and Secreted; RHE: Reconstructed human epidermis; RNA-seq: RNA Sequencing; RNS: Reactive Nitrogen Species; ROS: Reactive Oxygen Species; SEM: Scanning Electron Microscopy; SKALP: Skin-derived antileukoproteinase; TEER: Transepithelial Electrical Resistance; TEWL: trans-epidermal water loss; TGF-β1: Transforming Growth Factor beta 1; TIMPs: Tissue Inhibitors of Metalloproteinases; TLR2: Toll-like receptor 2; TNF-α: Tumor Necrosis Factor alpha; VEGFA: Vascular Endothelial Growth Factor A; ZO-1: Zonula Occludens-1.

### 5.1 Acute wound induction in skin models

A prerequisite for wound modeling is the establishment of full-thickness skin equivalents. Acute wounding strategies in such constructs have been widely used to study re-epithelialization, matrix remodeling, and keratinocyte–fibroblast interactions under healthy conditions.

A widely adopted method involves the mechanical induction of excisional wounds in HSEs, as seen in the model developed by Egles et al. (2010). In this system, normal human keratinocytes were seeded onto a collagen matrix embedded with dermal fibroblasts and cultured at ALI. After 7–10 days of maturation, the constructs were physically wounded and transferred to a secondary matrix, allowing keratinocytes to migrate over a fibroblast-populated substrate. Furthermore, fibroblasts preconditioned on denatured collagen significantly accelerated reepithelialization and enhanced basal keratinocyte proliferation, highlighting the importance of the fibroblast phenotype in modulating epidermal repair. Comparable wound-healing systems established by Laplante et al. (2001) and El Ghalbzouri et al. (2004) embed fibroblasts within dermal matrices over which stratified keratinocytes are layered. Following mechanical injury, these constructs recapitulate key aspects of native regenerative interactions, allowing stratified keratinocytes to migrate over an existing wound bed while preserving fibroblast–keratinocyte crosstalk. Such models provide a controlled platform for studying the dynamics of reepithelialization in response to direct tissue injury.

Alternative methods aim to reduce variability. A ring-exclusion strategy, for instance, generates standardized cell-free zones by preventing initial keratinocyte adhesion, after which keratinocytes migrate centripetally upon ring removal (Deshayes et al., 2018). This enables reproducible assessment of migration and early differentiation without mechanical disruption artifacts.

Acute wound models offer platforms for early-stage pharmacological investigation, as they enable controlled conditions for keratinocyte migration, fibroblast responses, and matrix remodeling. For example, wound models have shown that exogenous keratinocyte growth factor (KGF) and insulin-like growth factor-1 (IGF-1) enhance keratinocyte migration and viability, reinforcing the suitability of acute models for evaluating pro-migratory and pro-proliferative therapies (Stadelmann et al., 2025). Likewise, fibrin-rich 3D microenvironments within wounded HSEs recapitulate the coordinated sequence of keratinocyte migration, fibroblast recruitment, and granulation tissue formation, supporting the testing of agents that target myofibroblast differentiation, collagen deposition, and wound closure kinetics (Vilela De Sousa et al., 2023). In addition to biochemical stimuli, acute wounded skin equivalents also enable the testing of physical stimuli, such as pulsed electric fields, which have been shown to enhance actin reorganization and migration in dermal fibroblasts (Xiang et al., 2023).

However, their translational predictive value remains limited because they do not capture the pathological features that define non-healing wounds, such as fibroblast senescence, persistent cytokine exposure, impaired angiogenesis, excessive MMP activity, and altered macrophage polarization (Fernández-Guarino et al., 2023; Hofmann et al., 2023a). As a result, drug candidates that perform well in acute HSE assays may fail to demonstrate efficacy when tested under chronic wound–like conditions. Therefore, acute wound models are best positioned as the first step within tiered drug-screening pipelines.

### 5.2 Adding biological complexity: incorporation of adipose, vascular, and neural components

Most HSEs consist of a keratinocyte-based epidermis over a fibroblast-populated dermis, but chronic wound models may require additional cell types, including adipocytes, endothelial cells, and sensory neurons, to capture the complexity of subcutaneous tissue, vascular networks, and neurocutaneous interactions.

#### 5.2.1 Integration of subcutaneous tissue

The subcutaneous layer actively regulates epidermal barrier function and dermal remodeling through adipokine signaling. Incorporating adipose tissue into human skin equivalents alters gene expression programmes linked to ECM organization, keratinocyte differentiation, and metabolic regulation, while improving barrier function and epidermal architecture (Avelino et al., 2024). Multiple strategies have been explored to recreate this layer. One approach involves differentiating mesenchymal stem cells into mature adipocytes using inductive cocktails, though this method is time-, cost-, and material-intensive (Satija et al., 2007). Alternatively, mature adipocytes can be directly isolated from tissue, but their fragility and specific media requirements complicate co-culture (Huber et al., 2016). A more comprehensive strategy is the use of native adipose tissue, which introduces not only adipocytes but also adipose-derived stem cells, endothelial cells, smooth muscle cells, and macrophages, collectively enhancing the structural and functional complexity of the model (Ataç et al., 2013).

Complex constructs combining epidermal, dermal, and adipose compartments demonstrate the importance of this layer in disease modeling. For instance, diabetic skin equivalents containing healthy keratinocytes, diabetic fibroblasts, and a vascularized adipose compartment recapitulate hallmarks of diabetic wounds under hyperglycemia, including impaired re-epithelialization, reduced epidermal thickness, aberrant differentiation, elevated pro-inflammatory cytokines, oxidative stress, insulin resistance, and vascular leakage (Kim et al., 2021). Patient-derived lipoaspirates further advance this approach by providing heterogeneous cell populations, including mature adipocytes, endothelial cells, smooth muscle cells, and immune subsets, which can be maintained in long-term culture when embedded in biomaterial scaffolds (Abbott et al., 2016; Vidal et al., 2019).

#### 5.2.2 Integration of endothelial cells

Incorporating endothelial cells into dermal matrices promotes the formation of capillary-like structures, a step toward reproducing angiogenesis in human skin equivalents. These networks emerge through the combined action of endothelial cells, fibroblasts, and keratinocytes: fibroblasts deposit ECM and secrete angiogenic factors that support endothelial survival and organization, while keratinocytes contribute additional pro-angiogenic cues, including VEGF. Collagen-based hydrogels remain the most widely used scaffolds for generating vascularized constructs, yet they are often limited by matrix contraction, enzymatic degradation, and the instability or regression of vascular structures over time. To overcome these constraints, newer approaches employ alternative biomaterials, including fibrin, hyaluronic-acid scaffolds, human-derived decellularized ECM (dECM) (rich in native proteins and growth factors), and dECM-based bioinks, which provide more physiological architectures and improved support for microvascular organization (Ponec et al., 2004; Rimal et al., 2024). Despite progress, vascularized HSEs remain rudimentary compared with in vivo angiogenesis models such as the murine dorsal skinfold chamber or chick chorioallantoic membrane (Grambow et al., 2021). HSEs' endothelial networks still generate immature and short-lived endothelial networks that lack the sprouting, anastomosis, and remodeling characteristic of in vivo angiogenesis (Vailhé et al., 2001). Therefore, these models are better suited for investigating early endothelial behavior, cell–cell communication, and matrix interactions than for fully reproducing complex vascular morphogenesis.

Due to these limitations, recent innovations have focused on developing perfusable cultured platforms that can promote endothelial maturation and improve vascular stability. Strategies such as sacrificial-templated microchannels, including electrospun poly(3-hydroxybutyrate-*co*-3-hydroxyvalerate) (PHBV) combined with alginate fibers, can be endothelialized and incorporated into bilayered skin constructs (Dikici et al., 2020). Bioprinted perfusable networks have also provided additional stabilization advantages; for example, Mori et al. (2017) showed that perfused HUVEC-lined channels remain lumenized, preserve endothelial junctions, and improve nutrient delivery, enhancing dermal cell viability and epidermal stratification. Microfluidic skin-on-chip models further extend these capabilities by enabling immune-cell circulation and pathogen-induced vascular responses (Sun et al., 2022). Additionally, perfusion bioreactors have been shown to reduce vascular regression and improve endothelial survival under dynamic flow conditions (Helmedag et al., 2015).

#### 5.2.3 Incorporation of sensory neurons

Sensory neurons have gained interest as active regulators of wound repair, particularly through neuroepidermal and neuroimmune crosstalk. Innervated skin models have demonstrated that neuronal inputs accelerate wound closure, partly via secretion of neuropeptides such as substance P, which stimulates keratinocyte migration and proliferation through neurokinin-1 receptors. Inhibition of this pathway abrogates the effect, underscoring its functional relevance (Blais et al., 2014).

Building on this concept, Vidal et al. (2019) developed a tri-layered HSE that incorporated a neuronal layer to mimic neurocutaneous interactions. Their model utilized human induced neural stem cells (hiNSCs) embedded in a collagen hydrogel between the dermal and hypodermal compartments. Neuronal viability, confirmed by βIII-tubulin (TUJ1) staining, persisted for at least six weeks, with preserved morphology and distribution. Functionally, the inclusion of hiNSCs reduced pro-inflammatory markers (e.g., IL-6, RANTES, NOS2) compared to neuron-free controls, suggesting a modulatory role for neurons in dampening inflammation, a critical feature in chronic wounds, where neurogenic inflammation and sensory dysfunction impair healing. Despite the relevance of innervated HSEs for modeling chronic wounds (e.g., diabetic foot ulcers and pressure ulcers associated with neuropathy), no studies have yet applied these models specifically to chronic wound conditions.

From this section, we can verify that a unmet need in chronic wound modeling remains the development of integrated systems that can simultaneously incorporate vascularized adipose tissue and functional innervation. Technical barriers persist due to incompatible culture requirements between cell types. Neuronal cultures typically require neurotrophic factors, such as nerve growth factor (NGF), which may impair keratinocyte differentiation (Vidal et al., 2019). In contrast, adipocyte maintenance often necessitates serum-containing media that promote fibroblast overgrowth (Huber et al., 2016). The development of such integrated systems would require optimized media formulations that balance metabolic demands across neural, vascular, and epithelial compartments.

### 5.3 Supplementation with pathophysiological stimuli

#### 5.3.1 Hyperglycemia, AGEs, and hypoxia

The diabetic wound microenvironment is characterized by significant metabolic dysregulation, with hyperglycemia and advanced glycation end products (AGEs) as drivers for diabetic ulcers. In vitro studies using 2D cultures have demonstrated that high glucose impairs proliferation, migration, and angiogenic potential in endothelial cells, while promoting apoptosis and oxidative stress (Huang et al., 2019; Zhao et al., 2020). AGEs exacerbate this dysfunction by binding to receptors for AGEs (RAGE), activating NOX4, suppressing Nrf2-dependent antioxidant responses, and driving ferroptosis through disrupted iron and glutathione metabolism (Chen et al., 2024; Van Putte et al., 2016). In keratinocytes and fibroblasts, chronic high glucose exposure reduces migration, proliferation, and matrix remodeling (Kruse et al., 2016; Lan et al., 2008), with fibroblasts exhibiting the most pronounced transcriptional alterations (S. Zhang et al., 2021a).

Hypoxia further compounds metabolic stress. While hypoxia stimulates VEGF secretion across multiple skin-resident cell types, hyperglycemia selectively compromises fibroblast function, reducing the release of angiogenic and regenerative factors. When combined, hyperglycemia and hypoxia synergistically impair fibroblast-mediated repair, whereas adipose-derived stem cells retain relative resilience by maintaining VEGF and KGF secretion (Lafosse et al., 2016).

Importantly, impaired healing in diabetes is not solely attributable to metabolic cues. Immunological dysregulation, exemplified by an elevated neutrophil-to-lymphocyte ratio, frequently observed in individuals with type 2 diabetes mellitus, perpetuates chronic inflammation. Recent evidence suggests that this imbalance in immune cell populations may be driven less by hyperglycemia per se and more by persistent epigenetic reprogramming of circulating immune cells prior to their infiltration into the wound bed (Fernández-Guarino et al., 2023), reinforcing the need for models that integrate both metabolic and immune alterations.

In 3D in vitro skin models, hyperglycemia is rarely applied in isolation. Ozdogan et al. (2021), for example, developed a fully diabetic 3D human skin model comprising keratinocytes, fibroblasts, and endothelial cells derived from type II diabetic donors, and exposed the construct to high-glucose medium for nine days following ALI culture. While this approach simulated hyperglycemia, the model also incorporated pathologically relevant cell sources, which contributed to the observed wound healing impairment, such as incomplete re-epithelialization and altered fibroblast behavior. Similarly, Kim et al. (2021) engineered a skin construct comprising diabetic fibroblasts, diabetic preadipocytes, and a perfusable, vascularized subcutaneous tissue, which was maintained under high glucose. The pathological features reproduced in this model, including epidermal thinning, delayed wound closure, and inflammation, were the result of multiple overlapping diabetic cues rather than glucose alone. Thus, these studies illustrate that while glucose supplementation is a relevant component, its impact is most meaningful when integrated into multifactorial 3D models that more comprehensively represent the diabetic wound microenvironment.

#### 5.3.2 Pro-inflammatory cytokines exposure

Chronic wounds are sustained by a persistent inflammatory response driven by elevated levels of pro-inflammatory cytokines, including IL-1α, IL-1β, IL-6, IL-8, and TNF-α. These mediators, primarily released by activated neutrophils, macrophages, and platelets, perpetuate leukocyte recruitment and inflammatory signaling and impede progression into the proliferative phase of healing, and contribute to the chronicity of non-healing wounds (Zhao et al., 2016).

In vitro, pro-inflammatory environments can be recreated by supplementing engineered tissues with pro-inflammatory cytokines. Microfluidic skin-on-chip systems demonstrate how localized TNF-α exposure induces inflammatory responses across multiple cell types. For instance, perfusion of TNF-α through the fibroblast-containing dermal compartment upregulated IL-1β, IL-6, and IL-8 in the endothelial layer, disrupting tight junction proteins and increasing paracellular permeability (Wufuer et al., 2016). Similarly, wound-on-chip model embedding fibroblasts and endothelial cells in a 3D matrix recapitulates cytokine-induced loss of barrier integrity, elevated inflammatory signaling, and responsiveness to anti-inflammatory drugs such as dexamethasone (Biglari et al., 2019).

These models demonstrate the utility of cytokine supplementation in inducing controlled inflammatory states, thereby enabling mechanistic studies and pharmacological screening. However, this approach reproduces only a fraction of the complex immunological dysregulation of chronic wounds, which involves dynamic interactions among immune, stromal, and epithelial compartments under prolonged stress. Thus, while valuable for probing cytokine-mediated signaling, these systems fall short of fully capturing the persistence and heterogeneity of inflammation in non-healing wounds.

#### 5.3.3 Chronic wound fluid exposure

An alternative to supplementing cultures with defined stimuli is the use of wound exudates, which contain a mixture of pro-inflammatory cytokines (e.g., IL-1α, IL-1β, IL-6, IL-8, TNF-α) and proteolytic enzymes (MMP-1, MMP-2, MMP-8, MMP-9, MMP-13, uPA, neutrophil elastase, prolidase) characteristic of chronic wound microenvironment. Although some variations exist among wound types, such as increased MMP-1/TIMP-1 ratios in diabetic foot ulcers and elevated TIMP-1 in pressure ulcers, most components are shared across chronic wounds (Löffler et al., 2013).

HSE models have been used to study the distinct effects of acute wound fluid (AWF) and chronic wound fluid (CWF). While AWFs promote fibroblast viability, keratinocyte migration, and organized re-epithelialization, CWFs induce fibroblast apoptosis, keratinocyte hyperplasia, and matrix degradation, recapitulating hallmarks of chronicity (fibroblast apoptosis, keratinocyte hyperplasia, and collagen degradation) (Besser et al., 2017). Gene expression analyses confirm impaired fibroblast migration and disrupted matrix remodeling, consistent with delayed closure observed by hyperspectral imaging (Wahabzada et al., 2017).

Despite their physiological relevance, CWFs pose challenges. Proteolytic activity can degrade cytokines, such as IL-1β and TNF-α, during incubation, thereby reducing their bioactivity and potentially altering outcomes. Prolonged exposure may even paradoxically enhance fibroblast proliferation as cytotoxic components are inactivated (Burian et al., 2024). These findings display both the utility and the limitations of wound fluid supplementation: while capable of reproducing some aspects of the chronic microenvironment, batch variability and instability of bioactive molecules constrain reproducibility and predictive value.

### 5.4 Incorporation of chronic wound-derived cells

A significant advancement in the in vitro modeling of non-healing wounds is the incorporation of cells isolated directly from chronic wound tissues. Fibroblasts from chronic wounds exhibit reduced proliferative and migratory capacity (Wall et al., 2008), attenuated responsiveness to growth factors, and elevated MMP activity, leading to excessive ECM degradation. Keratinocytes from chronic wounds similarly display compromised proliferation and re-epithelialization potential (Yussof et al., 2012). Incorporating these patient-derived cells into 3D models helps retain critical aspects of the chronic wound phenotype, including aberrant signaling, impaired regeneration, and dysfunctional ECM remodeling.

Comparative studies highlight the impact of fibroblast dysfunction on wound repair. In HSEs, fibroblasts from diabetic foot ulcers lead to severely impaired re-epithelialization, reduced epidermal thickness, and disorganized architecture, whereas fibroblasts from non-ulcerated diabetic skin produce intermediate outcomes, and healthy fibroblasts enable more complete closure and stratification (Maione et al., 2015). These observations reinforce the notion that fibroblast pathology is central to the chronic wound phenotype.

Beyond fibroblast-focused models, full chronic wound cellular environments have been developed. Diabetic donor–derived keratinocytes, fibroblasts, and endothelial cells embedded in photocrosslinked gelatin hydrogels reproduce multiple hallmarks of diabetic wounds, including incomplete re-epithelialization, impaired vascular maturation, and altered fibroblast behavior under hyperglycemia (Ozdogan et al., 2021). Such constructs move closer to capturing the full cellular environment of chronic wounds.

### 5.5 Incorporation of immune cells

Immune cells are central regulators of wound repair, and their integration into 3D models represents an advancement for capturing the complexity of chronic inflammation. Key immune populations include Langerhans cells in the epidermis (Neagu et al., 2022b), dermal dendritic cells, macrophages, mast cells in perivascular regions, and T lymphocytes clustered in the superficial dermis (Tong et al., 2015), collectively forming specialized immunological niches. In chronic wounds, however, this balance is disrupted.

Diabetic ulcers are marked by persistent neutrophil infiltration, excessive release of proteases, ROS generation (Ellis et al., 2018), dysregulated NET formation (Fadini et al., 2016), and an elevated neutrophil-to-lymphocyte ratio, often driven by epigenetic reprogramming of immune cells rather than hyperglycemia alone (Fernández-Guarino et al., 2023). Additionally, hyperglycemia promotes a shift toward pro-inflammatory M1 macrophages (Ellis et al., 2018), accompanied by reduced recruitment of regulatory T cells and increased infiltration of Th17 cells (Fernández-Guarino et al., 2023). CD4^+^ T cell depletion and altered CD4/CD8 ratios further contribute to persistent immune activation (Loots et al., 1998). Venous ulcers similarly feature iron-laden macrophages locked in an M1 state, expressing high levels of iNOS, IL-12, and TNF-α, alongside reduced anti-inflammatory markers (Ellis et al., 2018; Zamboni et al., 2006). Hypoxia and secondary infections exacerbate macrophage dysfunction in both wound types (Ellis et al., 2018; Loots et al., 1998).

Co-culturing immune cells with skin-resident cells remains technically challenging due to incompatible culture requirements. For instance, macrophages lose viability in standard skin media, while macrophage-specific conditions impair epidermal differentiation (Griffoni et al., 2021). Advances in self-assembled skin substitutes with endogenous ECM have enabled macrophage survival and in situ monocyte-to-macrophage differentiation, maintaining functional wound responses (Smith et al., 2020). Diabetic-specific models embedding monocytes with diabetic fibroblasts further reproduce M1-like inflammatory phenotypes without disrupting epidermal stratification (Smith et al., 2021).

Lipoaspirate-based systems offer an alternative to integrate both adipose and immune fractions into engineered tissues, introducing lymphocytes, monocytes/macrophages, and other immune subsets that remain viable over the course of weeks of culture (Abbott et al., 2016; Vidal et al., 2019). Dynamic platforms extend these approaches. Wound-on-chip systems have demonstrated how M1 versus M2 macrophages differentially regulate cytokine production and vascularization. Specifically, M1 macrophages amplify pro-inflammatory cytokine secretion, while M2 macrophages enhance vascular network formation (Biglari et al., 2019). Composite models incorporating monocytes and pre-activated T cells capture complex cytokine profiles reminiscent of those induced by burns (Mulder et al., 2023).

Together, these advances underscore the potential of immune-integrated constructs to replicate the chronic inflammatory milieu of non-healing wounds. Yet, standardizing culture conditions to maintain diverse immune subsets alongside epithelial, stromal, and vascular compartments remains a critical barrier to fully exploiting these models for translational applications.

### 5.6 Skin infection mimicking in 3D wound models

Microbial colonization is a defining feature of chronic wounds, where polymicrobial consortia, dominated by *Staphylococcus aureus*, *Pseudomonas aeruginosa*, and β-hemolytic streptococci, impede re-epithelialization, sustain inflammation, and exacerbate tissue degradation (Zhao et al., 2013). Opportunistic pathogens, including ESKAPE pathogens (*Enterococcus* spp., *Klebsiella pneumoniae*, *Acinetobacter baumannii*, and *Enterobacter* spp.), *Proteus* spp., and coagulase-negative staphylococci, further complicate infections (Bowler et al., 2001). Anaerobes, such as *Peptoniphilus asaccharolyticus* and *Finegoldia magna*, have also been detected in deeper wound layers, which can worsen clinical outcomes. In addition, fungal species such as *Candida albicans* and *Aspergillus* spp. can form polymicrobial biofilms with bacteria, further enhancing antimicrobial resistance (Kalan and Grice, 2018; Uberoi et al., 2024).

Early infection models relied on ECM-based systems. Collagen matrices supplemented with simulated wound fluid supported biofilm formation and recapitulated antibiotic tolerance (Price et al., 2016; Werthén et al., 2010). 2D host cell-based assays provided further insight into pathogen-specific effects: keratinocyte apoptosis (Secor et al., 2011) or altered fibroblast cytokine secretion in strain- and mode-specific manners (Kirker et al., 2012). In this context, HSEs have emerged as more physiologically relevant platforms for infection studies. They replicate pathogen-specific invasion dynamics (Shepherd et al., 2009), distinguish commensals from pathogens (Holland et al., 2009, Holland et al., 2008), and capture a range of infection-induced changes, including in proteomic signatures (Havlikova et al., 2020), antimicrobial peptide expression (Haisma et al., 2013), and epithelial integrity (Holland et al., 2009, Holland et al., 2008).

Recent advances include HSEs colonized with polymicrobial biofilms, which better replicate clinical infections and enable the evaluation of antiseptic and antimicrobial treatments. Such models demonstrate the potential of reconstructed human skin for testing therapeutic interventions while preserving host–pathogen interactions (Brown et al., 2022; Charles et al., 2009). A complementary approach uses collagen-based wound models enriched with simulated wound fluid to support biofilm persistence and antibiotic resistance patterns seen in the clinic (Price et al., 2016). Together, these platforms not only demonstrate the value of infection-mimicking constructs but also reveal the need for systems that combine microbial, immune, and metabolic cues to capture the full complexity of chronic wound infections.

## 6 Pharmacological target investigation using 3D skin models

The strategies discussed in Section 5 show that advanced human skin models can mimic chronic wound pathology. However, their value for translation becomes clear when they are used to study drug mechanisms and test new treatments under conditions relevant to the human body. Therefore, Section 6 explores how different types of 3D human skin models enable researchers to investigate the specific pathways involved in non-healing wounds. Table 2 provides a summary of this section, listing the key features of each model, the mechanistic pathways that can be studied, and the related therapeutic targets or treatment strategies.

**Table 2:** Overview of pharmacological mechanisms investigated using Human skin equivalents (HSE) employed to model chronic wounds.

3D Skin Model characteristics	Mechanistic pathways interrogated	Representative therapeutic strategies/targets
Multicellular constructs: - HSEs with adipocytes; - HSEs with endothelial networks; - Neuron-integrated HSEs.	- Adipokine signaling (leptin, adiponectin, IL-6); - FGF–FGFR2 axis; - HIF-1α/VEGF; - ERK and AKT phosphorylation; - Ang1/Tie-2 stabilization; - Neurogenic inflammation; - Substance P/NK-1; TRPV1-mediated calcium signaling.	- ADSC-secretome therapies (Nrf2 exosomes); - FGF/FGFR2 activators; - Ang2 inhibitors; - Tie-2 modulators; - NK-1 receptor antagonists; - Neuromodulators; - Neuropeptide therapies (SP, CGRP, NPY, VIP); - TRP channel modulators.
HSEs supplemented with pathophysiological stimuli (hyperglycemia, AGEs, cytokines, chronic wound fluid).	- RAGE–NOX4 axis; - Nrf2/KEAP1 redox pathways; - Ferroptosis (iron metabolism, GPX4); - STAT3 and JAK1/2 activation (IL-6 loop); - MyD88–IRAK4–TRAF6 (IL-1 signaling); - NF-κB persistent activation; - Potease imbalance (MMP-1/8/9/13 vs TIMPs); - Plasmin–MMP cascades.	- Nrf2 activators; - RAGE inhibitors/soluble RAGE; - Antioxidant enzyme mimetics (SOD/GPX analogues); - Iron chelators; - GPX4 stabilizers; - JAK/STAT inhibitors; - IRAK4 inhibitors; - IKKβ/NF-κB blockers; - MMP inhibitors; - TIMP stabilizers; - Oxidative MMP-activation blockers.
HSEs integrated with chronic wound–derived cells (keratinocytes, fibroblasts, adipocytes, endothelial cells).	- p16/p21-driven senescence; - DDR activation; - SASP (NF-κB, C/EBPβ, p38 MAPK, JAK/STAT, mTOR); - Fibroblast mitochondrial ROS stress; - Myofibroblast dysfunction; - Impaired VEGF responsiveness; - Endothelial senescence.	- Senescence modulators; - SASP inhibitors; - Antioxidants; - Mitochondrial protectors; - Agents restoring fibroblast ECM synthesis; - MMP inhibitors; -Pro-angiogenic VEGF sensitizers; - Adipokine-modulating therapies.
HSEs containing immune cells (macrophages, neutrophils, T cells, dendritic cells).	- M1/M2 polarization dynamics; - Neutrophil chemotaxis, ROS, NETosis; - T-cell activation and transmigration; - Dendritic-cell cytokine production; - Immune-epithelial crosstalk.	- Macrophage-reprogramming agents (IL-4, IL-13, IL-10, PPAR-γ agonists); - Anti-NETosis agents (DNases, NET inhibitors); - Anti-IL-17 A; - PDE4 inhibitors; - Chemokine-engineered biologics (e.g., CCL20-LD); - Iimmunomodulatory biomaterials (cytokine-loaded, ROS-responsive, MMP-sequestering scaffolds).
Infection-integrated HSEs (bacteria, fungi, biofilms)	- Biofilm maturation; - Antimicrobial tolerance; - AMP alteration; - Infection-driven MMP upregulation; - Neutrophil immobilization; - NET accumulation; - Oxidative stress amplification; - Suppression of keratinocyte proliferation.	- Quorum-sensing inhibitors; - Antibiofilm peptides; - Enzymatic dispersal agents (DNases, proteases); - Nanoparticle antimicrobials; - Host-directed immunomodulators; - Combination therapies.

ADSC: adipose-derived stromal cells; AGEs: advanced glycation end-products; AMP: antimicrobial peptides; Ang1: Angiopoietin-1; CCL20-LD: C—C Motif Chemokine Ligand 20 Locked Dimer; CGRP: Calcitonin Gene-Related Peptide; DDR: DNA damage response; FGF: fibroblast growth factor; FGFR2: fibroblast growth factor receptor 2; GPX: Glutathione Peroxidase; GPX4: glutathione peroxidase 4; HIF-1α: hypoxia-inducible factor 1-alpha; IRAK4: interleukin-1 receptor–associated Kinase 4; JAK1/2: Janus Kinase 1 and 2; KEAP1: kelch-like ech-associated protein 1; MMP: matrix metalloproteinase; MyD88: myeloid differentiation primary response 88; NETs: Neutrophil Extracellular Traps; NOX4: NADPH Oxidase 4; NPY: Neuropeptide Y; Nrf2: nuclear factor erythroid 2–related factor 2; p38 MAPK: p38 Mitogen-Activated Protein Kinase; PDE4: Phosphodiesterase 4; RAGE: receptor for advanced glycation end-products; SASP: Senescence-Associated Secretory Phenotype; SOD: superoxide dismutase; SP: Substance P; STAT3: signal transducer and activator of transcription 3; Tie-2: tyrosine kinase with immunoglobulin-like and EGF-like domains 2; TIMP: tissue inhibitor of metalloproteinases; TNF-α: Tumor Necrosis Factor alpha; TRAF6: TNF receptor–associated factor 6; TRPV1: transient receptor potential vanilloid 1; VEGF: vascular endothelial growth factor; VIP: Vasoactive Intestinal Peptide.

An advantage of these complex models over traditional assays is their capacity to estimate drug pharmacokinetics and pharmacodynamics within a tissue context. By recreating physiological barriers, such as the stratified epidermis, dense extracellular matrix, and, in advanced iterations, perfusable vasculature, these systems modulate drug penetration, distribution, metabolism, and local bioavailability (Poumay and Coquette, 2007; Rimal et al., 2021). Consequently, they capture the concentration-effect relationships and spatial drug gradients that determine therapeutic success in human skin.

The following subsections detail how specific model features directly enable this pharmacological interrogation. For instance, vascularized constructs allow the study of dynamic drug transport and clearance (Mori et al., 2017; Sundar et al., 2022), while immune-integrated models reveal how leukocytes actively modulate drug stability and target engagement (Y. Li et al., 2022; Smith et al., 2021). By evaluating drug candidates under conditions that mimic the pathological wound microenvironment, these platforms help discard compounds whose efficacy in simple assays relies on unrealistically high or sustained local concentrations that are unachievable in vivo. Conversely, they identify promising candidates by informing the design of delivery systems (e.g., protease-resistant formulations, biomaterial-based sustained release) that ensure adequate tissue retention and robust target engagement (Boateng and Catanzano, 2015).

### 6.1 Enhancing pharmacological relevance through multicellular skin constructs

The integration of adipocytes, endothelial cells, and neurons into engineered wound models, as described in Section 5.2, creates platforms for investigating therapies that target the crosstalk between these compartments and the healing process.

Adipose-containing HSEs provide platforms to assess therapeutic strategies aimed at improving wound repair through modulation of adipose–epidermal–dermal interactions. These models are suitable for evaluating interventions such as adipose-derived stromal cell (ADSC) secretomes or exosomes engineered to overexpress Nrf2, which have been shown in vivo to enhance re-epithelization and angiogenesis by reducing oxidative stress and inflammation (Li et al., 2018). They also allow investigation of biologically relevant pathways like adipocyte lipolysis and adipocyte-to-myofibroblast transition, which contribute to macrophage recruitment and angiogenesis (Shook et al., 2020).

Vascularized skin equivalents and skin-on-chip platforms offer a mechanistic tool for studying pro-angiogenic and vasomodulatory drug candidates. By integrating patient-derived vascular cells and controlled flow, these systems enable the evaluation of targets involved in chronic wound angiogenesis. These can include for instance the fibroblast growth factor (FGF)–fibroblast growth factor receptor 2 (FGFR2) axis, whose activation drives endothelial proliferation and neovascularization (Sim et al., 2025); downstream effectors such as ERK and AKT, whose phosphorylation status correlates with angiogenic capacity (Kim et al., 2000; Mavria et al., 2006); and the Angiopoietin-1/Tie-2 signaling pathway, involved in the vascular stabilization and inflammation resolution (Kim et al., 2000).

Innerved HSEs are particularly relevant for the study of diabetic wounds, where neuropathy disrupts sensory signaling and neurogenic inflammation. These models provide a platform for investigating several pharmacological classes. Among these, important examples include neurokinin-1 (NK−1) receptor antagonists, which block Substance P-mediated inflammatory signaling, fibroblast proliferation, and angiogenesis (Li et al., 2023). The platform also facilitates research into neuromodulators that regulate neuroimmune responses under conditions of metabolic stress (Al Mamun et al., 2025). Additionally, neuropeptide-based regenerative therapies, such as those utilizing Substance P or calcitonin gene-related peptide (CGRP), can be evaluated for their ability to accelerate re-epithelialization and promote a pro-repair macrophage polarization (Xing et al., 2024). Furthermore, innervated HSEs allow for the study of modulators targeting Transient Receptor Potential (TRP) channels, including TRPV1, which are key regulators of nociception, neurogenic inflammation, and cellular migration in cutaneous cells (Bagood and Isseroff, 2021).

### 6.2 Enhancing pharmacological relevance through pathophysiological supplementation

Models supplemented with the diabetic metabolic, inflammatory and proteolytic cues detailed in Section 5.3 enable the mechanistic evaluation of therapeutic designed to counteract these scpecific stressors.

Models exposed to hyperglycemia and AGEs creates a biochemical landscape in which redox imbalance, mitochondrial dysfunction, and impaired cytoprotective signaling become experimentally accessible (Chen et al., 2024; Van Putte et al., 2016; J. Zhang et al., 2021b). This includes Nrf2-activating compounds, redox-sensitive cysteine inhibitors (e.g., targeting KEAP1 or ERK), and antioxidant enzyme mimetics (e.g., SOD/GPX analogues) (Li et al., 2025). AGE-enriched constructs further enable mechanistic interrogation of the AGEs–RAGE axis, supporting the evaluation of small-molecule RAGE antagonists, soluble RAGE decoys, or agents that suppress downstream NADPH oxidase–dependent ROS production (Zhou et al., 2024). Additionally, because metabolic stress in these models perturbs intracellular iron handling, lipid peroxidation, and glutathione metabolism, they are well-suited for assessing ferroptosis-directed interventions, such as iron chelators (Ramadoss et al., 2023) and GPX4-stabilizing molecules (Friedmann Angeli et al., 2014).

Cytokine-conditioned 3D skin models and skin-on-chip systems, which recapitulate persistent inflammatory signaling, are ideal for testing immunomodulatory therapies. These platforms allow for the evaluation of small-molecule kinase inhibitors targeting JAKs or IRAK4, as well as agents that supress NFκB activation (e.g., IKKβ inhibitors), given the central role of the MyD88–IRAK4–TRAF6, JAK/STAT, and NF-κB pathways in sustaining inflammation (Guo et al., 2024; Gupta et al., 2010; Zarrin et al., 2021).

CWF-supplemented constructs, which reproduce a protease-rich microenvironment, serve as platforms for evaluating MMP inhibitors, TIMP stabilizers, and therapies designed to preserve ECM integrity. Furthermore, because MMP induction is controlled by upstream inflammatory mediators and oxidative stress, these models also support testing of AP-1/NF-κB pathway blockers, antioxidants, and MMP-resistant growth factor formulations (Krishnaswamy et al., 2017; Löffler et al., 2013).

Beyond small molecules, 3D skin models can be employed for testing peptide-based therapeutics. Recent innovations include engineered peptides designed to resist proteolytic degradation in the chronic wound milieu. For instance, protease-resistant antimicrobial peptides such as D-TN6 exhibit broad-spectrum antibacterial and antifungal activity, effectively reducing bacterial burden and controlling infection in wound environments (Kocagoz et al., 2025). Another strategy involves peptides that combine antimicrobial and immunomodulatory properties. Host defense peptides, such as cathelicidins and defensins, promote leukocyte recruitment, regulate pro- and anti-inflammatory cytokine production, stimulate angiogenesis, and enhance keratinocyte migration and proliferation, accelerating wound closure and preventing excessive inflammation (Martell et al., 2021). Furthermore, peptide-based therapeutics targeting specific innate immune receptors, particularly members of the formyl peptide receptor (FPR) family, have demonstrated efficacy in dampening neutrophil-driven inflammation and NETosis in preclinical models. Activation of pro-resolving pathways such as FPR2/ALX by annexin A1-derived peptides has been shown to reprogram neutrophil function toward a pro-resolutive phenotype, thereby contributing to the interruption of chronic inflammatory cycles (Ansari et al., 2021). The microenvironments of 3D skin constructs can thereby provide a platform to evaluate the potential of these next-generation peptide therapies within a human-relevant context.

### 6.3 Enhancing pharmacological relevance through chronic wound–derived cells

As outlined in Section 5.4, 3D models incorporating chronic wound-derived keratinocytes, fibroblasts, adipocytes, or endothelial cells retain pathological traits such as senescence and impaired function. The evaluation of therapeutic targets in these models can be approached through two strategies.

First, several molecular pathways can be interrogated independently of the specific chronic wound cell type, as they represent conserved hallmarks of chronic wound pathology. These include interventions targeting cellular senescence programs regulated by p16INK4a, p21CIP, and the DNA damage response (DDR); modulation of the senescence-associated secretory phenotype (SASP) driven by NF-κB, C/EBPβ, p38 MAPK, Jak2/STAT3, and mTOR; and restoration of redox homeostasis to counteract ROS-mediated senescence in fibroblasts and endothelial cells (Wei et al., 2022).

Second, a more specific strategy involves assessments of therapeutic targets tailored to the principal chronic wound-derived cell type populating the 3D skin model. This cell-type-specific approach allows for targeted interventions. For instance, in chronic wound-derived keratinocytes, the model permits the study of pathways involved in defective re-epithelialization, including impaired proliferation and migration caused by persistent DDR activation, p16/p21 overexpression, and SASP-mediated paracrine inhibition (Pastar et al., 2023; Wei et al., 2022). Chronic wound-derived fibroblast models allow for evaluating modulators of myofibroblast dysfunction, excessive MMP production, impaired ECM synthesis, SASP-driven secondary senescence, and signaling disruptions that arise from TNF-α, IL-1 family cytokines, and ROS-mediated mitochondrial stress (Kita et al., 2024; Pastar et al., 2023; Ukaegbu et al., 2025; Wei et al., 2022). Chronic wound-derived adipocyte models enable exploration of therapies acting on metabolic stress–induced senescence, and impaired adipokine secretion (Cooper et al., 2021; Wei et al., 2022). Finally, chronic wound-derived endothelial cell models provide a platform for the mechanistic evaluation of interventions aimed at key dysfunctions in chronic wound healing. These include targeting endothelial cell senescence, which is associated with a loss of angiogenic capacity and reduced responsiveness to VEGF; addressing ROS-driven endothelial dysfunction; and modulating the pronounced SASP that actively impedes neovascularization (Pastar et al., 2023; Ukaegbu et al., 2025; Wei et al., 2022).

### 6.4 Enhancing pharmacological relevance through immune cells

The immune-integrated constructs described in Section 5.5, which replicate the dysregulation of chronic inflammation, are valuable for investigating immunomodulatory therapies.

First, these constructs offer a controlled and reproducible platform for studying macrophage polarization dynamics under wound-like conditions. For instance, researchers can apply candidate macrophage-reprogramming agents, such as cytokines (IL-4, IL-13, IL-10) or small-molecule agonists of transcriptional regulators (e.g., PPAR-γ agonists), to monitor the phenotypic shift from a pro-inflammatory M1 state toward pro-regenerative M2 subtypes. These effects can be evaluated across multiple levels, including marker expression, functional readouts (e.g., phagocytosis, matrix deposition, collagen synthesis), and downstream effects on other resident skin cells, such as fibroblasts, keratinocytes, and adipocytes. Indeed, 3D in vitro models have already been employed to demonstrate the specific role of M2-like macrophage subtypes in facilitating tissue repair (Sapudom et al., 2021).

Second, immune-integrated models enable testing of therapies aimed at modulating neutrophil-mediated pathology. Although neutrophils have a short lifespan and are challenging to maintain in long-term cultures, several groups have successfully incorporated neutrophils into 3D dermal matrices (R. Li et al., 2022) and skin-on-chip systems (Kim et al., 2019) for short-term studies. In 3D collagen-based matrices, neutrophils exhibit improved viability, preserved multilobulated nuclear morphology, enhanced ROS production, increased chemotaxis, and NET formation in response to PMA, LPS, IL-1β, and TNF-α, effectively recapitulating in vivo–like NETosis dynamics (R. Li et al., 2022). In parallel, skin-on-chip platforms using human biopsies have demonstrated neutrophil recruitment toward infected tissue within 4–6 h, enabling quantitative assessment of chemotaxis, extravasation, and infection-dependent activation (Kim et al., 2019). These co-cultures enable the mechanistic investigation of early neutrophil-driven responses, providing a foundation for studying downstream processes, such as NET formation, ROS-mediated tissue injury, and the impact of anti-NETosis therapies.

Third, by incorporating lymphocytes such as T cells into advanced skin models (Hollstein et al., 2025; Ren et al., 2021) or by integrating additional immune subsets, including dendritic cell surrogates (Hölken et al., 2023), it becomes feasible to explore modulators of adaptive immunity in human-relevant systems. In immunocompetent HSEs, primary or Peripheral Blood Mononuclear Cells (PBMC)-derived T cells have been successfully incorporated and shown to respond to inflammatory cytokines, chemokine cues, and therapeutic agents such as anti-IL-17 A or Phosphodiesterase-4 (PDE4) inhibitors (Hollstein et al., 2025). Likewise, skin-on-chip platforms enable the quantitative investigation of T-cell transmigration, chemotaxis, and allow the testing of targeted immunomodulators, such as the engineered CCL20-LD (C—C motif chemokine ligand 20 Locked Dimer) (Ren et al., 2021). Moreover, skin models containing functional dendritic cells exhibit cytokine production (IL-6, IL-8, TNF-α, and IL-1β) and drug-responsive immune activation, providing an additional entry point to assess how immune-driven signals influence downstream tissue remodeling and epithelial responses (Hölken et al., 2023).

Finally, and importantly for the translational potential, immune-integrated 3D skin constructs are ideally suited for evaluating immunomodulatory biomaterials: scaffolds, hydrogels, or nano/microstructured delivery systems designed to modulate the wound immune microenvironment. In this context, biomaterials may be engineered to (i) present immunomodulatory signals (e.g., cytokines, chemokines), (ii) sequester excessive pro-inflammatory mediators (e.g., TNF-α, MMPs), (iii) respond to pathological cues such as high ROS or elevated MMP activity by releasing anti-inflammatory drugs or antioxidants, or (iv) deliver immune-modulating cells (e.g., mesenchymal stromal cells, regulatory immune cells) (Las Heras et al., 2024; Wang et al., 2025).

### 6.5 Enhancing pharmacological relevance through skin infection

Infection-integrated 3D models, as detailed in Section 5.6, replicate polymicrobial colonization and biofilm-driven inflammation, offering translational value for antimicrobial discovery. HSEs colonized with *S. aureus*, *P. aeruginosa*, and Candida spp. reproduce strain-specific cytotoxicity, epithelial barrier breakdown, altered antimicrobial peptide profiles, and induction of host- and microbe-derived proteases (Haisma et al., 2013; Holland et al., 2008; Shepherd et al., 2009).

Biofilm-rich constructs enable the testing of diverse therapeutic strategies, including quorum-sensing inhibitors, antibiofilm peptides, enzymatic dispersal agents (e.g., DNases, proteases, glycosidases), topical formulated antimicrobials, host-directed immunomodulators, and combination therapies. They also provide a platform to investigate pathogen–host interactions, such as biofilm-induced MMP upregulation, neutrophil immobilization and NET accumulation, oxidative stress amplification, keratinocyte proliferation arrest, and suppression of re-epithelialization (Kim et al., 2019; Price et al., 2016; Werthén et al., 2010).

## 7 Pathophysiological relevance in drug efficacy testing

The predictive validity of preclinical drug testing depends on the pathophysiological relevance of the 3D in vitro skin models used. Models that inadequately recapitulate key features of chronic wounds risk generating misleading efficacy data. For example, anti-inflammatory compounds tested in simplified keratinocyte-fibroblast co-cultures lacking immune cells (Wufuer et al., 2016) often show exaggerated benefits, as they omit the persistent neutrophil infiltration, macrophage-driven inflammation, and self-sustaining cytokine networks that characterize non-healing wounds in vivo (Ellis et al., 2018). Likewise, antimicrobial studies conducted in planktonic or single-species biofilm models (Haisma et al., 2013) may underestimate clinical resistance by neglecting the protective ECM, metabolic cooperation, and interspecies signaling of polymicrobial infections (Brown et al., 2022).

Therapies addressing multifactorial pathogenesis, such as immunomodulators, biofilm-targeting agents, or stem cell therapies, are particularly sensitive to the limitations of oversimplified models. Evaluating macrophage-polarizing strategies, for instance, requires systems that reproduce the immune disequilibrium of chronic wounds, including M1-skewed macrophage populations, impaired M2 transition, and inadequate T-cell–mediated resolution (Smith et al., 2021), instead of relying on monocultures that do not sustain chronic inflammatory circuits. Similarly, antibiofilm agents must be tested in systems combining colonization with host responses, such as excessive MMP activity and impaired epithelial migration (Kadam et al., 2019), since some antimicrobials reduce bacterial load while paradoxically hindering repair (Daeschlein, 2013). For combination therapies, e.g., antimicrobials paired with growth factors or pro-angiogenic agents, outcomes depend on the balance between infection control and tissue regeneration, further emphasizing the need for models that integrate multiple pathological dimensions.

At the same time, reductionist models remain valuable for investigating isolated mechanisms, which are specific to certain types of cells. For instance, keratinocyte migration assays utilizing fibroblasts isolated from chronic wounds (Maione et al., 2015) offer an efficient platform for screening pro-migratory compounds, as this process can be studied without the full complexity of immune cell interactions or microbial components. Similarly, MMP activity and ECM remodeling can be evaluated in fibroblast-populated matrices exposed to chronic wound fluid (Besser et al., 2017), given the role of fibroblasts in ECM turnover. Likewise, studies focusing on impaired angiogenesis may employ endothelialized constructs subjected to hyperglycemic and hypoxic conditions (Lafosse et al., 2016) without necessarily incorporating infectious elements that would complicate the interpretation of vascular responses.

Taken together, these considerations argue for a tiered, mechanism-informed testing strategy rather than a one-size-fits-all approach. In the early phases of drug discovery, high-throughput, reductionist models can be utilized to map pathway engagement, rank candidate molecules, and distinguish between on-target and off-target effects with a favorable signal-to-noise ratio. Subsequent validation in progressively more complex 3D skin systems, incorporating metabolic stressors, chronic wound–derived cells, immune components, and infection when appropriate, allows researchers to challenge candidate therapies under conditions that more closely approximate to chronic wound microenvironment. Only through such a stratified pipeline, in which the complexity of the model is matched to the therapeutic mechanism of action, can preclinical findings gain sufficient predictive power.

## 8 Current limitations and future perspectives

The human skin models reviewed herein represent an advance over traditional 2D cultures and animal models for chronic wound research. By integrating pathological stimuli, patient-derived cells, immune compartments, and microbial biofilms, they provide a mechanistic resolution for therapeutic evaluation. However, despite these significant strides, critical gaps remain that limit their full predictive power and clinical translatability.

A primary challenge lies in sustaining integrated pathophysiology over biologically relevant timescales. While models successfully incorporate individual hallmarks, such as hyperglycemia, cytokine exposure, or biofilm colonization, these conditions are often applied transiently. True chronicity, however, involves the persistent, low-grade dysregulation of multiple systems over weeks to months. Maintaining this state in vitro is technically demanding: patient-derived cells frequently lose pathological phenotypes after a few passages, immune cells exhibit limited viability in standard skin media, and complex co-cultures struggle with incompatible metabolic requirements (Griffoni et al., 2021; Ozdogan et al., 2021). Consequently, most models capture a “snapshot” of dysfunction rather than the dynamic progression of a non-healing wound. Furthermore, challenges related to standardization and scalability present significant technical and economic barriers to the broader implementation of complex in vitro models in drug discovery. The reliance on patient-specific or primary cells, biologically complex matrices, and specialized bioreactor-based platforms leads to high inter- and intra-experimental variability and increases operational costs, limiting reproducibility and scalability with high-throughput screening pipelines required for efficient drug development (Baran, 2022; Ekert et al., 2020).

Besides, the reductionist compartmentalization of disease drivers remains a limitation. As detailed in 5, 6, models excel at isolating specific mechanisms (e.g., immune polarization, infection), but no existing platform fully integrates the triad of metabolic dysregulation, immune dysfunction, and polymicrobial persistence within a single system. For instance, biofilm-infected models (Brown et al., 2022) rarely incorporate the specific immune dysregulation (e.g., elevated neutrophil-to-lymphocyte ratio, impaired macrophage transition) and metabolic stress (e.g., AGE-RAGE axis) associated with diabetic ulcers. This disconnect limits the evaluation of combination therapies designed to concurrently address infection, inflammation, and impaired healing.

Future efforts should prioritize the development of chronic wound models that integrate patient-matched immune cells (e.g., monocytes, T cells), stromal cells (fibroblasts, adipocytes), and endothelial networks within a tunable ECM. The goal is to recapitulate the failed resolution of inflammation, where dysregulated crosstalk between senescent fibroblasts, M1-skewed macrophages, and impaired T-cell function perpetuates tissue damage (Ellis et al., 2018; Smith et al., 2021). Advancing engineering strategies for sustained chronicity will be equally relevant. This includes leveraging organ-on-chip and dynamic bioreactor technologies to maintain long-term culture viability and to introduce physiological gradients (e.g., oxygen, cytokines). Microfluidic platforms with integrated biosensors could further enable real-time monitoring of metabolic activity, protease levels, and biofilm burden, providing kinetic data on therapeutic response (Rimal et al., 2021; Sriram et al., 2018).

It is worth mentioning that a promising frontier lies in integrating artificial intelligence (AI) and machine learning (ML) analytics in experimental studies. Recent data demonstrate that ML and deep learning approaches can extract clinically relevant information from digital wound images and tissue-derived data, enabling automated wound detection and monitoring in real-world settings (Cassidy et al., 2023). Beyond classification, hybrid AI models combining deep neural networks with regression algorithms can predict clinically meaningful outcomes, such as time-to-healing by quantifying wound area and tissue composition (Kolli et al., 2024). In parallel, interpretable deep learning applied to histological images has identified collagen fiber organization as a robust biomarker of wound healing dynamics and delayed healing (He et al., 2025). Together, these advances demonstrate the potential of AI in analyze complex outputs from advanced skin models that can be used to interpret experimental data and to stream-line experiments.

For these models to achieve regulatory acceptance as predictive tools, a clear pathway linking in vitro endpoints to clinical human-based outcomes must be established. An opening step is standardization of protocols for cell sourcing, matrix composition, and quantitative readouts to ensure reproducibility, a prerequisite for any regulatory submission (Baran, 2022; Ekert et al., 2020). Encouragingly, a strong precedent exists: RHE models are already internationally validated and accepted for skin irritation and corrosion testing under OECD guidelines (TG 439, 431) (OECD, 2021, OECD, 2019). This establishes a framework for qualifying more complex wound models.

Building on this, a strategic, two-phase approach can accelerate adoption: first, employing standardized skin models for safety and local tolerance assessments, aligning with existing guidance (e.g., ISO 10993-23) (ISO, 2021); and second, pursuing mechanistic qualification of wound-specific models for defined contexts-of-use (e.g., diabetic or infected ulcers), validated against clinical datasets. This direction is reinforced by a global regulatory shift toward New Approach Methodologies (NAMs), evidenced by initiatives like the FDA's ISTAND pilot program (U.S. Food and Drug Administration, 2020) and the FDA Modernization Act 2.0 (United States Congress, 2022), which encourages non-animal evidence for safety and efficacy. Finally, a critical step will be establishing clear correlation metrics between in vitro model endpoints (e.g., gene expression signatures, cytokine profiles, closure kinetics) and clinical outcomes. This validation is critical for transitioning these platforms from research tools into regulatory-accepted alternatives to animal testing.

## CRediT authorship contribution statement

**Regina Gomes Daré:** Writing – review & editing, Writing – original draft, Conceptualization. **Luciana B. Lopes:** Writing – review & editing. **Alke Petri-Fink:** Writing – review & editing. **Barbara Rothen-Rutishauser:** Writing – review & editing, Conceptualization.

## Funding

This work was supported by 10.13039/501100003071Adolphe Merkle Foundation and 10.13039/501100001807Fundação de Amparo à Pesquisa do Estado de São Paulo (FAPESP; 2024/08941-3; 2018/13877-1).

## Declaration of competing interest

The authors declare that they have no known competing financial interests or personal relationships that could have appeared to influence the work reported in this paper.

## Data Availability

No data was used for the research described in the article.
